# A self-assembling luminescent lanthanide molecular nanoparticle with potential for live cell imaging†
†Electronic supplementary information (ESI) available: Experimental procedures and X-ray crystallographic data for **1–4** in CIF format. CCDC 1031249–1031252. For ESI and crystallographic data in CIF or other electronic format see DOI: 10.1039/c8sc00650d


**DOI:** 10.1039/c8sc00650d

**Published:** 2018-04-26

**Authors:** Xiaoping Yang, Shiqing Wang, Yali Zhang, Guang Liang, Ting Zhu, Lijie Zhang, Shaoming Huang, Desmond Schipper, Richard A. Jones

**Affiliations:** a College of Chemistry and Materials Engineering , Wenzhou University , Wenzhou 325035 , China . Email: xpyang@wzu.edu.cn ; Email: smhuang@wzu.edu.cn; b Zhejiang Key Laboratory of Carbon Materials , Wenzhou 325035 , China; c Chemical Biology Research Center , School of Pharmaceutical Science , Wenzhou Medical University , Wenzhou 325035 , China . Email: wzmcliangguang@163.com; d The University of Texas at Austin , Department of Chemistry and Biochemistry , 1 University Station A5300 , Austin , Texas 78712 , USA . Email: rajones@cm.utexas.edu

## Abstract

Four luminescent 32-metal Cd–Tb nanoclusters, [Tb_8_Cd_24_(L^1^)_12_(OAc)_48_] (**1**), [Tb_8_Cd_24_(L^2^)_12_(OAc)_48_] (**2**), [Tb_8_Cd_24_(L^3^)_12_(OAc)_48_] (**3**) and [Tb_8_Cd_24_(L^2^)_12_(1,4-BDC)_4_(OAc)_38_(OH)_2_] (**4**), were constructed from three specially designed chain-like Schiff base ligands H_2_L^1–3^ with flexible carbon–carbon backbones containing 5, 6 and 10 methylene units, respectively. The clusters exhibit drum-like structures and can be imaged using transmission electron microscopy (TEM). In addition to the Schiff base ligands (the primary energy transfer donors), four 1,4-BDC bridging units were successfully introduced into the structure of **4**. In addition to providing increased structural stability, the 1,4-BDC units act as secondary energy transfer donors providing extra energy for lanthanide luminescence, which results in improved luminescence properties when compared to those of the related Cd–Ln nanoclusters without 1,4-BDC units. *In vitro* investigations on **4** with SGC and PANC cancer cells revealed an accumulation of the molecular nanoparticles in the cells, as confirmed by confocal microscopy. The cytotoxicity of **4** toward the SGC and PANC cells is moderate (IC_50_ values of **4** lie in the range of 15–60 μM). ICP-MS analysis reveals that cellular uptakes of **4** in 1000 SGC and PANC cells after treatment for 3 hours are 0.0094 pmol and 0.015 pmol, respectively.

## Abstract 2

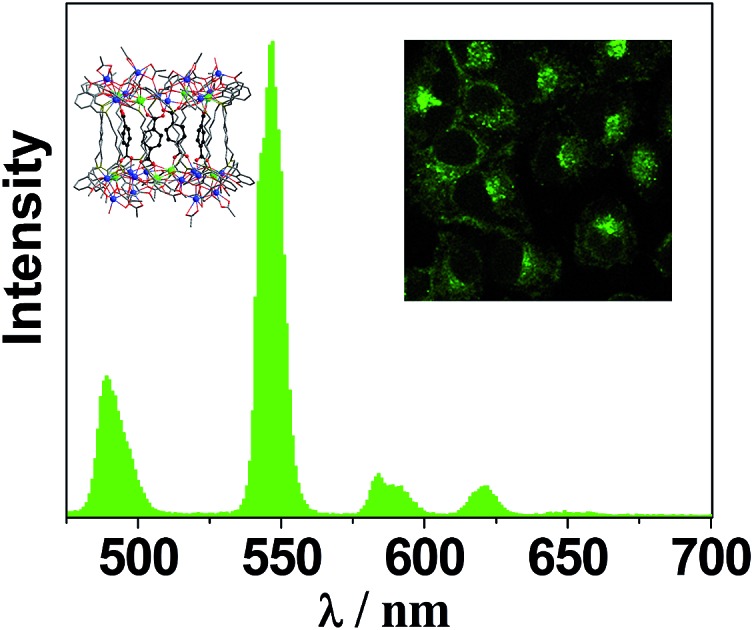
A 32-metal Cd–Tb nanocluster with enhanced visible luminescence was constructed by the introduction of energy transfer donors (1,4-BDC) for potential bioimaging applications.

## Introduction

“Nanodiagnostics” involves the use of engineered nanomaterials that can be biofunctionalized with target-specific molecules of interest, allowing ultra-sensitive detection. For example, luminescent nanoparticles are being widely investigated as cellular probes in the field of cell biology and biochemistry to get a better understanding of the structure and function of biological systems through methods that involve minimal perturbation of the system.^1^ The existing classes of probes in widespread use, such as fluorescent organic dyes, and fluorescent proteins and nanoparticles (*i.e.* quantum dots (QDs) and lanthanide-based nanoparticles), have shown great promise, but their utility is generally limited by small Stokes' shifts, short luminescence lifetimes, and broad and formless emission profiles.^2^

Recently, advances in time resolved microscopy have stimulated interest in lanthanide(iii) species (Ln = Sm, Eu, Tb, Dy, and Yb) as cell imaging agents.^3^ Most reports have so far been focused on the use of mononuclear lanthanide complexes as cellular probes due to their rational design.^3^ However, the lanthanide luminescence of such mononuclear complexes is susceptible to quenching effects caused by water or hydroxyl groups,^4^ which can quench the lanthanide luminescence through the vibrational mode of the OH-group. High-nuclearity 4f and d–4f nanoclusters are currently of interest not only due to their often aesthetically stunning molecular architectures but also their remarkable physical and chemical properties.^5^ With more organic ligands, the lanthanide ions may be enclosed in the cluster structures of high-nuclearity complexes, which can help to improve their luminescence properties by shielding the lanthanide centers from the outside solvent environment. The surface morphology and size of cellular probes may affect their biological activity. High-nuclearity lanthanide-based clusters exhibit a well-defined size and composition. They are much larger than mononuclear lanthanide complexes but smaller than QDs and lanthanide-based nanoparticles.^6^ Recently, Roesky *et al.* first reported the cell imaging properties of luminescent pentadecanuclear lanthanide nanoclusters.^7^ For d–4f clusters, light-absorbing d-block metal chromophores (*i.e.* Pt^II^,^8^ Ru^II^,^9^ Zn^II^,^10^ Cr^II^,^11^ and Cd^II^ (ref. 12)) can be used as sensitizers for visible and NIR luminescence from Ln(iii) centers following ligand → f and d → f energy-transfers. Many reports have so far been focused on polymeric 3d–4f clusters, such as Cu/Ln,^13^ Mn/Ln^14^ and Ni/Ln^15^ cluster systems, in order to study their magnetic properties as single-molecule magnets. However, high-nuclearity d–f systems with luminescence properties have received much less attention, especially with respect to their imaging applications in living systems. To the best of our knowledge, high-nuclearity d–4f nanoclusters have hitherto not been used for any biological application (see page 390 of ref. 3a).

**Scheme 1:**
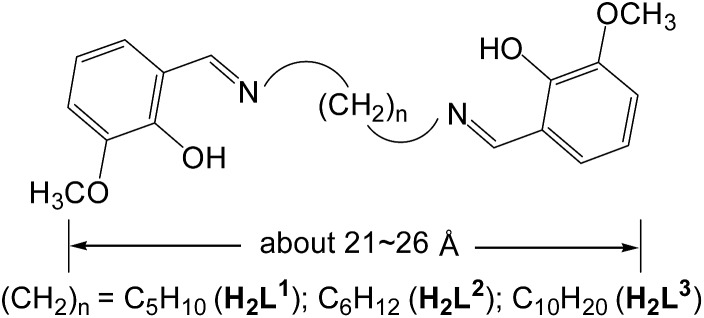
Flexible long-chain ligands H_2_L^1–3^.

Schiff base metal complexes have been investigated with regard to their potential as cellular localization probes.^16^ Salen-type Schiff base ligands (Scheme 1) with carbon–carbon (–CH_2_–CH_2_–) backbones capable of relatively free rotation have also been employed to synthesize d–f heteronuclear clusters.^17^ Typically, the (CH_2_)_*n*_ chain lengths of the ligands can affect the structures of the d–f clusters.^18^ In this work we report the use of three chain-like Schiff base ligands, H_2_L^1–3^ (Scheme 1), which have flexible carbon–carbon backbones containing 5, 6 and 10 methylene units, respectively. Reactions of H_2_L^1–3^ with Cd(OAc)_2_·4H_2_O and Ln(OAc)_3_·4H_2_O resulted in three 32-metal Cd–Tb nanodrum-like clusters, [Tb_8_Cd_24_(L^1^)_12_(OAc)_48_] (**1**), [Tb_8_Cd_24_(L^2^)_12_(OAc)_48_] (**2**) and [Tb_8_Cd_24_(L^3^)_12_(OAc)_48_] (**3**). Interestingly, despite the differences in the chain length of the three ligands, **1–3** have similar drum-like structures, which are not affected by the (CH_2_)_*n*_ chain lengths of the ligands.^19^ However, due to the differences in chain lengths, the specific molecular dimensions of the clusters are different, most notably in terms of the length of each of the nanodrums. We were naturally curious as to how these differences could be explored by further modifications to the molecular architectures of the drums. With this in mind we investigated the introduction of a second, carefully chosen, multidentate ligand. We have now discovered that the addition of 1,4-BDC (1,4-benzenedicarboxylate) to the reaction mixture of **2** leads to the formation of the 32-metal drum-like nanocluster [Tb_8_Cd_24_(L^2^)_12_(1,4-BDC)_4_(OAc)_38_(OH)_2_] (**4**), in which four 1,4-BDC bridging units are successfully introduced into the *inside* of the nanosize drum. The 1,4-BDC bridging units not only help to stabilize the drum-like structure of **4** but also appear to provide extra energy for lanthanide luminescence, resulting in superior luminescence properties of **4** compared to **1–3**.^19^ The Cd–Tb cluster **4** was also investigated to evaluate its potential for use as a luminescent probe in live cell imaging. To the best of our knowledge, this is the first report on the application of a high-nuclearity d–4f nanocluster as a bioimaging cellular probe.

## Results and discussion

### Synthesis and characterization

**Fig. 1:**
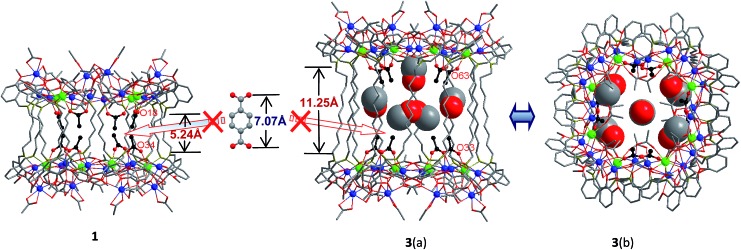
Views of the crystal structures of **1** and **3** (viewed along the *a*-axis (a) and *c*-axis (b) with enclosed solvents) (Tb^3+^: green; Cd^2+^: blue): their nano-drum-like structures are either too short or too high for the introduction of 1,4-BDC units.

**Fig. 2:**
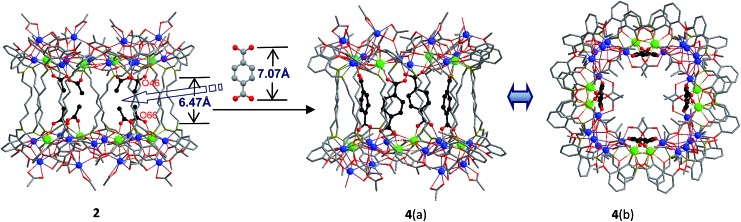
Views of the crystal structures of **2**, and **4** (viewed along the *a*-axis (a) and *c*-axis (b)) (Tb^3+^: green; Cd^2+^: blue).

Reactions of H_2_L^1–3^ with Cd(OAc)_2_·4H_2_O and Tb(OAc)_3_·4H_2_O in refluxing MeOH/EtOH produced yellow solutions from which the Cd–Tb nanoclusters **1–3** were isolated as pale yellow crystalline solids. As shown in Fig. 1 and 2, **1–3** have similar 32-metal nano-drum-like structures with two rings of Cd_12_Tb_4_ linked by 12 Schiff base ligands. Each Cd_12_Tb_4_ ring includes 24 OAc^–^ ions to balance the charge of the cluster. Two views of the crystal structure of **3** are shown in Fig. 1. The view of **3**(a) is essentially a side-on view while **3**(b) is a view looking down onto the top of the drum. Formed by the longest H_2_L^3^, the nanosize drum of **3** has a larger internal space than **1** and **2**, and some guest molecules such as MeOH and H_2_O are enclosed within its internal void (Fig. 1). During the course of our studies with chain-like Schiff base ligands H_2_L^1–3^ we have found that [Ln_8_Cd_24_L_12_(OAc)_48_] analogues (L = L^1–3^; Ln = Nd^3+^, Pr^3+^, Sm^3+^, Dy^3+^, Yb^3+^ and Lu^3+^) may also be isolated using similar experimental conditions. Thus, differences in ionic radii among the 4f ions do not result in significant changes in the drum-like structures. The Cd–Tb clusters **1–3** show much stronger visible lanthanide luminescence than their analogues such as those with Pr^3+^, Sm^3+^ and Dy^3+^ ions.

A very noteworthy common feature of these structures is the presence of eight OAc^–^ anions (drawn in black in Fig. 1 and 2), which are located on the inside of the nanodrums. This feature led us to the question of whether they could be replaced with related groups, for example, replacing two OAc units for one 1,4-BDC unit. With this possibility in mind we investigated the addition of 1,4-BDC to the reaction mixtures of **1–3**. For **1** and **3** no new complexes were isolated. However, in the case of **2**, the eight internal OAc^–^ anions are replaced by four 1,4-BDC units, resulting in the formation of **4**. Two views of the crystal structure of **4** are shown in Fig. 2 with the four 1,4-BDC units drawn in black. The new bonding situation in **4** thus features two Cd_12_Tb_4_ rings linked not only by 12 Schiff base ligands, but also the four 1,4-BDC units. It seems reasonable to assume that the increased binding between the two Cd_12_Tb_4_ rings would increase the overall thermodynamic stability of the nano-drum-like structure.^20^ Consistent with this is the decomposition point of **4** which is significantly higher than that of **2** (210 °C *vs.* 182 °C).

Although the precise mechanism of formation of the nanoclusters is not currently known the structural data do provide clues pertaining to the formation of **4**, produced from the reaction of 1,4-BDC with the reaction mixture of **2**, and why no reaction appears to take place in the case of **1** and **3**. The structures of **1–3** all contain long-chain Schiff base ligands which display “linear” configurations. Thus the separations of the two Cd_8_Tb_4_ based ends of each drum are dictated by the (CH_2_)_*n*_ chain length of H_2_L^1–3^. This results in the distances between OAc^–^ units at the ends of each drum increasing successively in the order **1**, **2**, and **3**. For example, the O–O distances of two separated OAc^–^ ions are 5.24 Å (O(18)–O(34)), 6.47 Å (O(46)–O(66)) and 11.25 Å (O(33)–O(63)) for **1–3**, respectively (Fig. 1 and 2). The O–O distance (6.47 Å) in **2** is close to the length of 1,4-BDC (7.07 Å) and thus 1,4-BDC fits the gap created by the absence of two OAc^–^ units. The analogous distances in **1** and **3** are either too short or too long for the replacement. These results indicate that the drum-like structure formed by H_2_L^2^ is suitable for the introduction of 1,4-BDC units, compared with those formed by H_2_L^1,3^.

**Fig. 3:**
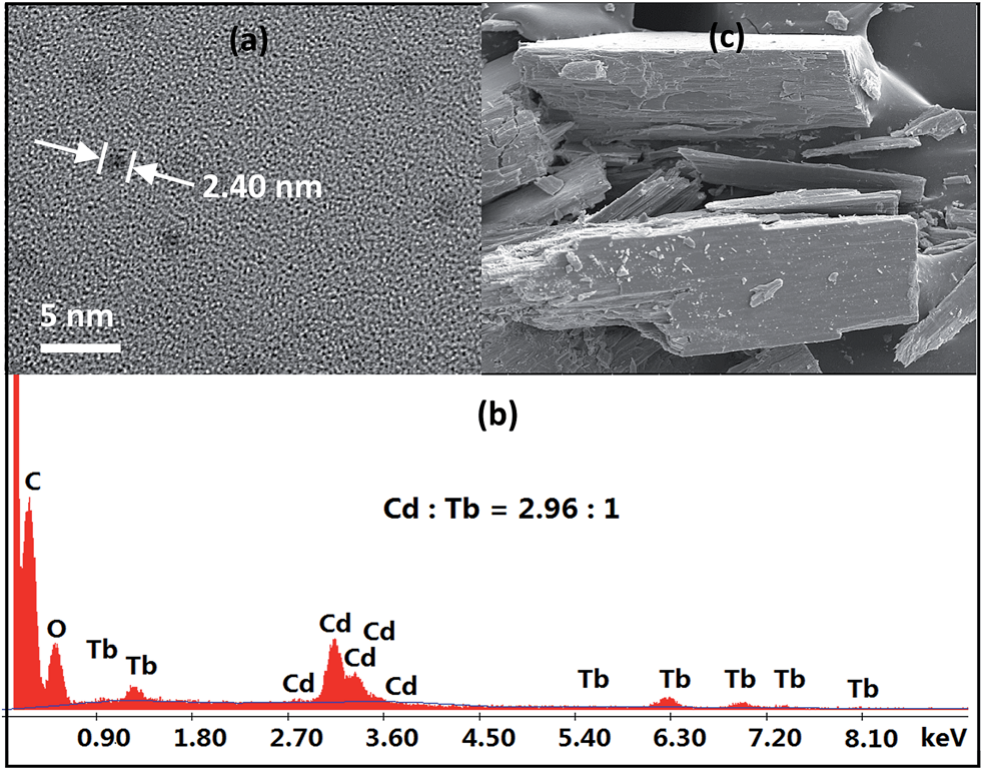
TEM (a) and SEM (c) images, and EDX (b) spectrum of **4**.

As revealed by X-ray structural data, **1–4** are all of nanoscale proportions. The molecular dimensions are approximately 20 × 24 × 24 Å for **1**, 23 × 24 × 24 Å for **2** and **4**, and 29 × 24 × 24 Å for **3**, which are much larger than those of most other lanthanide-based clusters with Schiff base ligands reported thus far. These dimensions have enabled us to obtain images of these molecular nanoparticles using transmission electron microscopy (TEM, Fig. 3a). Dilute solutions of **4** in MeCN were brought into contact with a Cu grid and the solvent carefully evaporated under vacuum. The TEM images obtained show uniform nanoparticles with diameters measuring approximately 2.40 nm, which corresponds well with the diameter of the 16-metal ring end of the drum found in the crystal structure. Energy dispersive X-ray spectroscopy (EDX) analysis of **4** indicates that the molar ratio of Cd/Tb is about 3 (Fig. 3b), in agreement with the crystal structure. These results indicate that the nanocluster retains its unique molecular structure in solution. The panoramic scanning electron microscopy (SEM) image in Fig. 3c shows the polycrystalline nature of **4**.

### Luminescence properties

**Fig. 4:**
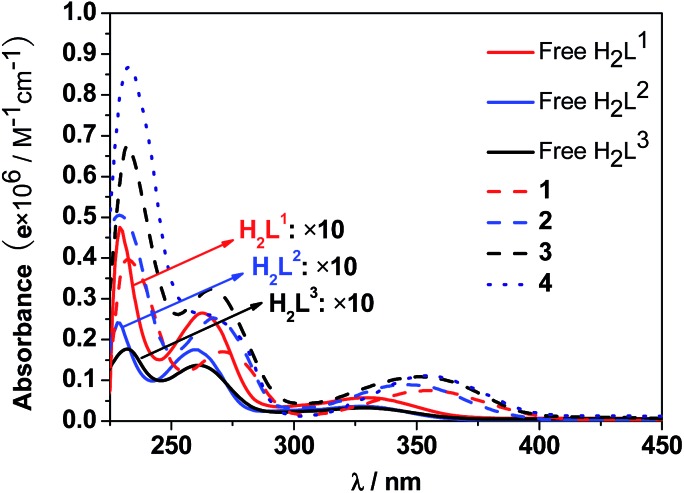
UV-Vis spectra of the free H_2_L^1–3^ and Cd–Tb clusters **1–4** in CH_3_CN (*C* = 10^–8^ to 10^–7^ M).

**Fig. 5:**
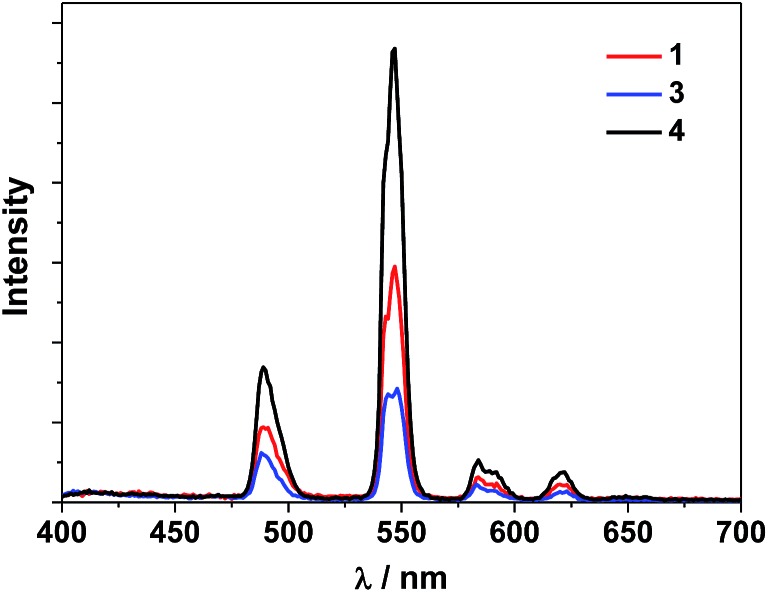
Visible emission spectra of Tb(iii) nanoclusters **1**, **3** and **4** in CH_3_CN (*λ*_ex_ = 340 nm).

The UV-Vis spectra of the free H_2_L^1–3^ and Cd–Tb clusters are shown in Fig. 4. The absorption bands of the free ligands H_2_L^1–3^ are red-shifted upon co-ordination to metal ions in the Cd–Ln nanoclusters. The absorptions of all nanoclusters are over 10 times stronger than those of their free ligands, which is advantageous for the ligand-center to absorb energy for sensitizing the lanthanide luminescence. The luminescence properties of **1–4** were studied in CH_3_CN. Upon excitation of the ligand-centered absorption bands (ESI Fig. S2†), **1–4** show the visible emission bands of Tb^3+^ (^5^D_4_ → ^7^F_*n*_ transitions, *n* = 6, 5, 4 and 3) (Fig. 5). The fluorescence quantum yields (*Φ*_em_) of **1–4** in CH_3_CN are 0.242, 0.230, 0.181 and 0.357, respectively. Thus, with four 1,4-BDC chromophores acting as energy transfer donors for lanthanide luminescence, the quantum yield of **4** is significantly higher than those of **1–3**. In **4**, four enclosed 1,4-BDC groups provide extra energy for lanthanide luminescence, and also occupy the internal space in the drum and thus help to protect the lanthanide ions from solvent molecules, which may quench the lanthanide luminescence through the vibrational modes of XH-groups (X = O or C).^21^ In addition, the more rigid structure of **4**, created by the presence of the four 1,4-BDC groups, may also contribute to the improvement of its luminescence properties.^22^

### Biological studies

**Fig. 6:**
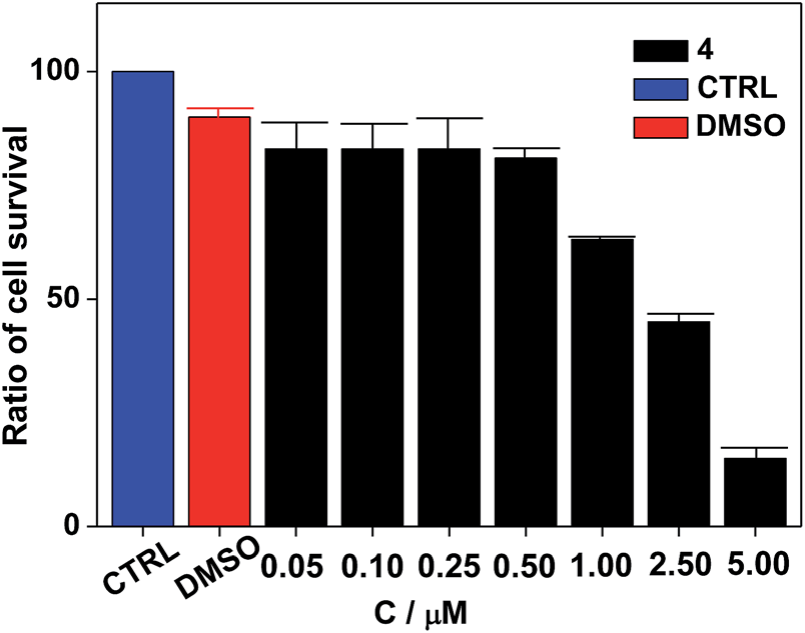
Dose responsive cell proliferation curves of SGC cells.

**Fig. 7:**
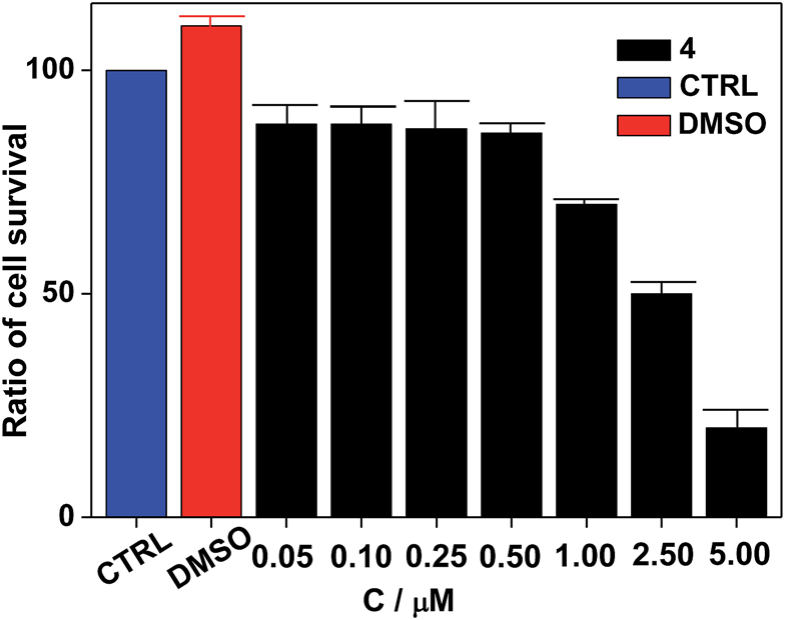
Dose responsive cell proliferation curves of PANC cells.

Nanocluster **4** was tested to evaluate its potential for use as a luminescent probe for cell imaging. To determine the inherent cytotoxicity of **4**, MTT assays were conducted using SGC and PANC cancer cells. As shown in Fig. 6 and 7, nanocluster **4** displayed an IC_50_ value of 2.1 ± 0.2 μM for SGC cells. A similar IC_50_ value of approximately 2.5 ± 0.5 μM was obtained for PANC cells. When corrected for the stoichiometries of **4** within the assembled nano-drum (8 Tb, 24 Cd, and 12 L^2^), the IC_50_ values of **4** lie in the range of 15–60 μM. These values are indicative of moderate to mild cytotoxicity and comparable to those of Eu(iii) and Tb(iii) molecular compounds that can be used as agents for cell imaging.^3^,^7^ To further understand the origin of the inherent cytotoxicity of **4**, the cytotoxicities of H_2_L^2^, Cd(OAc)_2_·2H_2_O and Tb(OAc)_3_·4H_2_O were also determined by MTT assays (ESI Fig. S3†). The H_2_L^2^ ligand and Cd(OAc)_2_·2H_2_O provided IC_50_ values of 37.5 ± 3.9 and 7.7 ± 1.2 μM for SGC cells, respectively, while Tb(OAc)_3_·4H_2_O displayed no inhibition of cell proliferation at the concentrations tested. Comparison of the cytotoxicity of **4** with those of these components that form assembled nano-drums suggests that the cytotoxic effects primarily originate from the Cd and ligand (L^2^). This is consistent with observations that the nature of the antenna (the Cd/L in the nano-drum of **4**) is the most important feature that controls toxicity in lanthanide bioprobes.^3^

**Fig. 8:**
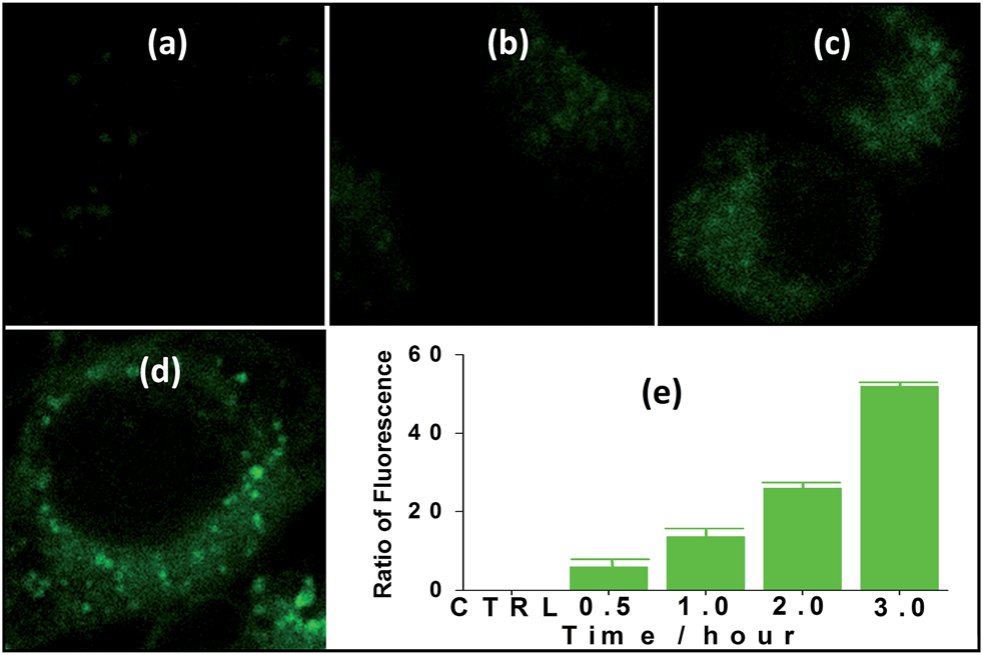
Fluorescence microscopy photographs of SGC cells treated with **4** (*c* = 250 nM) for 30 min (a), 1 h (b), 2 h (c) and 3 h (d), and fluorescence assay following treatment for different time periods (e).

**Fig. 9:**
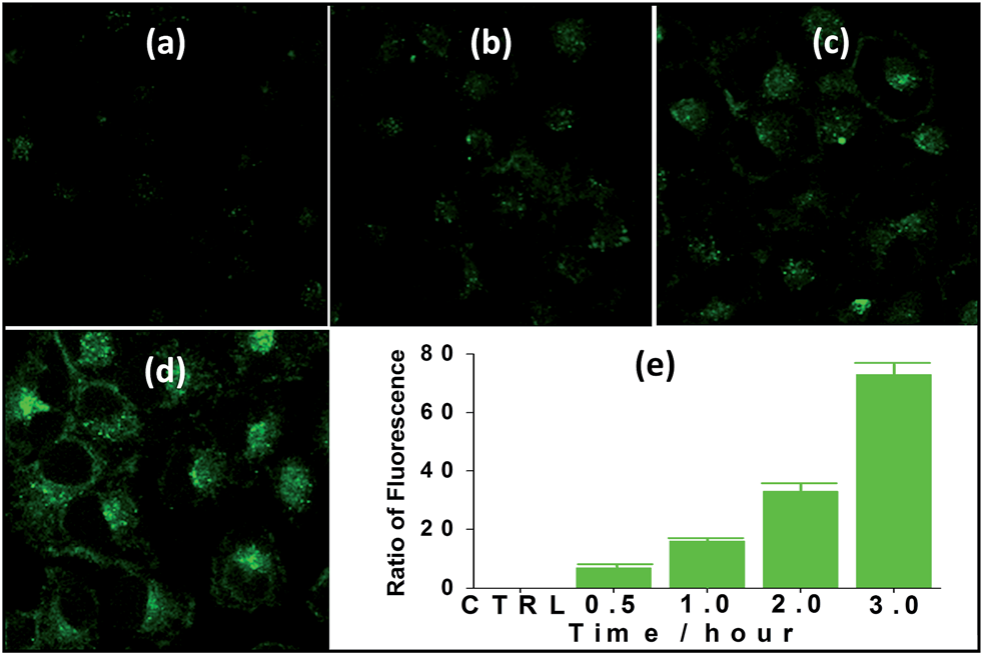
Fluorescence microscopy photographs of PANC cells treated with **4** (*c* = 50 nM) for 30 min (a), 1 h (b), 2 h (c) and 3 h (d), and fluorescence assay following treatment for different time periods (e).

**Fig. 10:**
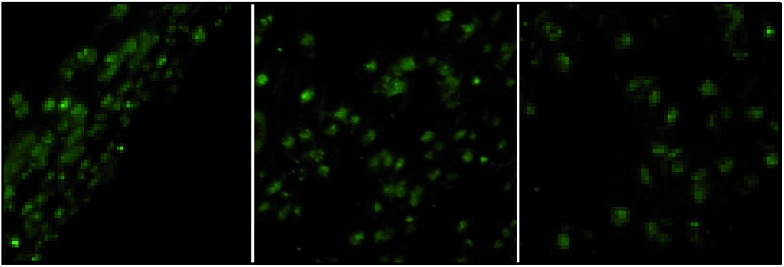
The multi-layered scanning photographs (viewed from different directions) of PANC cells treated with **4** (*c* = 1 nM) for 3 hours.

The suitability of **4** for use in bioimaging applications was evaluated using confocal microscopy. As shown in Fig. 8 and 9, upon excitation of the ligand-centered absorption bands (*λ*_ex_ = 340–380 nm), green fluorescence was observed in SGC cells and PANC cells treated with **4**, while no emission was found in the control group. The fluorescence intensity gradually increased in step with the increase of treatment time and concentration of **4** (Fig. 8 and 9, and ESI Fig. S4 and S5†). Multi-layered scanning of the cells clearly shows the presence of **4** in the interior of the cells (Fig. 10). The uptakes of the cluster in both cells were determined by ICP-MS analysis after treatment with **4** (the concentrations of **4** are 250 nM and 50 nM for SGC and PANC cells, respectively). The results indicate that, after treatment for 3 hours, 0.0094 pmol and 0.015 pmol of **4** are found in 1000 SGC and PANC cells, respectively (ESI Fig. S6†). Green fluorescence was not observed in SGC cells or PANC cells treated with the free Schiff base ligand (H_2_L^2^) or Tb(OAc)_3_·4H_2_O under the same experimental conditions.

## Experimental

### Materials and methods

Metal salts and other solvents were purchased from Aldrich and used directly without further purification. The SGC and PANC cancer cells were purchased from American Type Culture Collection (Manassas, VA). The Schiff-base ligands H_2_L^1–3^ were prepared according to well-established procedures.^23^ All reactions were performed under dry oxygen-free dinitrogen atmospheres using standard Schlenk techniques. Physical measurements: NMR: VARIAN UNITY-plus 600 spectrometer (^1^H, 600 MHz) at 298 K; powder XRD: SMART APE II DUO; IR: FTIR-650 spectrometer; melting points were obtained in sealed glass capillaries under dinitrogen and are uncorrected. Elemental analyses (C, H, and N) were carried out on an EA1112 elemental analyzer. Transmission electron microscopy (TEM) images were recorded on a JEOL JEM-1200EX transmission electron microscope. Field emission scanning electron microscopy (FESEM) images were recorded on a Nova NanoSEM 200 scanning electron microscope. Absorption spectra were obtained on a UV-3600 spectrophotometer, while excitation and emission spectra were recorded on a QuantaMaster PTI fluorimeter. Fluorescence quantum yields were determined by using quinine sulfate (*Φ* = 0.546 in 0.5 mol dm^–3^ H_2_SO_4_) as a standard for Tb^3+^ complexes.^24^

### Synthesis of [Tb_8_Cd_24_(L^1^)_12_(OAc)_48_] (**1**)

Cd(OAc)_2_·2H_2_O (0.60 mmol, 0.1595 g), Tb(OAc)_3_·4H_2_O (0.20 mmol, 0.0816 g) and H_2_L^1^ (0.30 mmol, 0.1113 g) were dissolved in MeOH (30 mL) at room temperature, and Et_3_N (0.60 mmol in MeOH 10 mL) was added. The resulting solution was stirred and heated under reflux for 20 min. It was allowed to cool and then filtered. Diethyl ether was allowed to diffuse slowly into the filtrate at room temperature and pale yellow crystals were obtained after one week. The crystals were collected by filtration, washed with MeOH and dried in air. Yield: 0.0905 g (30%). Mp > 185 °C (dec.). Elemental analysis: found: C, 36.02; H, 4.59; N, 2.63%. Calc. for C_348_H_432_Cd_24_N_24_O_144_Tb_8_(MeOH)_12_(H_2_O)_23_: C, 36.59; H, 4.25; N, 2.85%. IR (cm^–1^): 3430 (s), 2934 (s), 2855 (s), 1640 (s), 1550 (s), 1467 (s), 1304 (s), 1215 (s), 1076 (m), 1010 (m), 958 (w), 854 (w), 739 (m), 675 (w), 612 (w).

### Synthesis of [Tb_8_Cd_24_(L^2^)_12_(OAc)_48_] (**2**)

The procedure was the same as that for **1** except using H_2_L^2^ (0.30 mmol, 0.1155 g). Pale yellow single crystals of **2** were formed after one week. Yield: 0.1599 g (51%). Mp > 182 °C (dec.). Elemental analysis: found: C, 36.35; H, 4.99; N, 2.51%. Calc. for C_360_H_464_Cd_24_N_24_O_144_Tb_8_(MeOH)_17_(H_2_O)_16_: C, 36.98; H, 4.61; N, 2.75%. IR (cm^–1^): 3417 (m), 2921 (m), 2855 (m), 1633 (s), 1573 (s), 1464 (s), 1212 (m), 1079 (m), 1013 (w), 960 (w), 850 (w), 743 (m), 671(w), 615 (w).

### Synthesis of [Tb_8_Cd_24_(L^3^)_12_(OAc)_48_] (**3**)

The procedure was the same as that for **1** except using H_2_L^3^ (0.30 mmol, 0.1323 g). Pale yellow single crystals of **3** were formed after one week. Yield: 0.1186 g (37%). Mp > 191 °C (dec.). Elemental analysis: found: C, 38.87; H, 5.52; N, 2.33%. Calc. for C_408_H_552_Cd_24_N_24_O_144_Tb_8_(MeOH)_21_(H_2_O)_18_: C, 39.42; H, 5.15; N, 2.57%. IR (cm^–1^): 3331 (s), 2927 (s), 2835 (s), 1636 (s), 1575 (s), 1480 (s), 1341 (m), 1301 (m), 1238 (m), 1212 (m), 1079 (w), 1016 (w), 961 (w), 852 (w), 743 (m), 676 (m), 613 (w).

### Synthesis of [Tb_8_Cd_24_(L^2^)_12_(1,4-BDC)_4_(OAc)_38_(OH)_2_] (**4**)

Cd(OAc)_2_·2H_2_O (0.60 mmol, 0.1595 g), Tb(OAc)_3_·4H_2_O (0.20 mmol, 0.0816 g) and H_2_L^2^ (0.30 mmol, 0.1155 g) were dissolved in MeOH (30 mL) at room temperature, and Et_3_N (0.60 mmol in MeOH 10 mL) was added. The resulting solution was stirred and heated under reflux for 20 min. Then 1,4-BDC (1,4-benzenedicarboxylic acid) (0.0249 g, 0.15 mmol) and a solution of NaOH (5 mL of a 0.06 M solution in DI) were added. The solution was stirred and heated under reflux for 30 min. It was allowed to cool and then filtered. Diethyl ether was allowed to diffuse slowly into the filtrate at room temperature and pale yellow crystals were obtained after two weeks. The crystals were filtered off and washed with MeOH (5 mL). Yield (based on Tb(OAc)_3_·4H_2_O): 0.0859 g (28%). Mp > 210 °C (dec.). Elemental analysis: found: C, 38.50; H, 4.01; N, 2.56%. Calc. for C_372_H_444_Cd_24_N_24_O_142_Tb_8_(MeOH)_11_(H_2_O)_10_: C, 38.22; H, 4.22; N, 2.79%. IR (cm^–1^): 3424 (s), 2921 (s), 2848 (s), 1633 (s), 1573 (s), 1440 (s), 1406 (s), 1301 (m), 1215 (s), 1079 (m), 1016 (w), 960 (w), 847 (w), 736 (s), 675 (m).

### Cytotoxicity assays

The cytotoxicity profiles of nanocluster **4**, the H_2_L^2^ ligand, Cd(OAc)_2_·2H_2_O and Tb(OAc)_3_·4H_2_O were obtained by MTT (3-[4,5-dimethylthiazol-2-yl]-2,5-diphenyltetrazolium bromide) assays using SGC cells and PANC cells. SGC cells and PANC cells were plated on 96-well plates (3000 cells in 100 μL media per plate) and maintained at 37 °C in 5% CO_2_-humidified air for 24 hours along with each compound. After 24 hours, MTT (5 mg mL^–1^) workup of the cells in each well was conducted for 4 hours at 37 °C. The MTT was aspirated and DMSO (150 μL) was added to each well. The absorbance at 490 nm was read by means of a plate reader. Each assay was performed in triplicate, the mean value was calculated, and the results are reported as percent of control.

### Live cell imaging

SGC cells and PANC cells were plated on 35 mm plates (5000 cells in 1 mL media per plate) and maintained at 37 °C in 5% CO_2_-humidified air for 24 hours. They were treated with different doses of **4** for different time periods, and then washed with PBS (3×). The cells were fixed in 4% paraformaldehyde for 15 minutes and then washed with PBS (3×). The cells were imaged using confocal microscopy (Nikon, Tokyo, Japan). A combination of excitation filter DAPI-DM400 (EX 340–380 nm) and emission filter FITC (BA 515–555 nm) was used to obtain the cell images.

ICP-MS analysis for cellular uptake: SGC and PANC cells were co-cultured with **4** for 3 hours, harvested, and then treated with 60% HNO_3_ for over 24 hours at room temperature to ensure complete digestion. The samples were diluted with double-distilled water to give 10 mL solutions with 2% HNO_3_. The concentrations of Tb(iii) in the cells were determined using an inductively coupled plasma mass spectrometer (ICAP Qc, Thermo Fisher, USA), to afford the absolute Tb(iii) content per 1000 cells associated with the cell number. A standard curve was made for the quantitative determination. The values are the average ± SD of three independent experiments.

### Crystallography

Data were collected on a Smart APEX CCD diffractometer with graphite monochromated Mo-Kα radiation (*λ* = 0.71073 Å) at 190 K. The dataset was corrected for absorption based on multiple scans and reduced using standard methods. Data reduction was performed using DENZO-SMN.^25^ The structures were solved by direct methods and refined anisotropically using full-matrix least-squares methods with the SHELX 97 program package.^26^ Coordinates of the non-hydrogen atoms were refined anisotropically, while hydrogen atoms were included in the calculation isotropically but not refined. Neutral atom scattering factors were taken from Cromer and Waber.^27^ Selected bond lengths and angles are given in Tables S1–S4.†

#### **1**  

C_366_H_508_Cd_24_N_24_O_178_Tb_8_, tetragonal, space group *P*4/*n*, *a* = 30.0936(7), *b* = 30.0936(7), *c* = 39.3554(13) Å, *α* = 90°, *β* = 90°, *γ* = 90°, *V* = 35 641.2(17) Å^3^, *Z* = 2, *D*_c_ = 1.124 g cm^–3^, *μ*(Mo-Kα) = 1.539 mm^–1^, *F*(000) = 11 936, and *T* = 190 K. *R*_1_ = 0.0639 and w*R*_2_ = 0.1850 for 30 698 independent reflections with a goodness-of-fit of 0.912.

#### **2**  

C_386_H_560_Cd_24_N_24_O_190_Tb_8_, orthorhombic, space group *Fddd*, *a* = 54.777(11), *b* = 61.944(12), *c* = 79.935(16) Å, *α* = 90°, *β* = 90°, *γ* = 90°, *V* = 271 224(94) Å^3^, *Z* = 4, *D*_c_ = 1.229 g cm^–3^, *μ*(Mo-Kα) = 1.622 mm^–1^, *F*(000) = 99 776, and *T* = 190 K. *R*_1_ = 0.1275 and w*R*_2_ = 0.3051 for 57 932 independent reflections with a goodness-of-fit of 1.034.

#### **3**  

C_428_H_632_Cd_24_N_24_O_171_Tb_8_, orthorhombic, space group *Fddd*, *a* = 54.621(11), *b* = 61.479(12), *c* = 100.21(2) Å, *α* = 90°, *β* = 90°, *γ* = 90°, *V* = 336 505(99) Å^3^, *Z* = 4, *D*_c_ = 1.012 g cm^–3^, *μ*(Mo-Kα) = 1.307 mm^–1^, *F*(000) = 102 546, and *T* = 190 K. *R*_1_ = 0.0959 and w*R*_2_ = 0.2256 for 72 594 independent reflections with a goodness-of-fit of 1.081.

#### **4**  

C_386_H_500_Cd_24_N_24_O_177_Tb_8_, tetragonal, space group *P*4*n*2, *a* = 42.806(6), *b* = 42.806(6), *c* = 37.886(8) Å, *α* = 90°, *β* = 90°, *γ* = 90°, *V* = 69 419(23) Å^3^, *Z* = 4, *D*_c_ = 1.175 g cm^–3^, *μ*(Mo-Kα) = 1.582 mm^–1^, *F*(000) = 24 288, and *T* = 190 K. *R*_1_ = 0.0892 and w*R*_2_ = 0.2151 for 59 138 independent reflections with a goodness-of-fit of 1.102.

## Conclusions

In conclusion, we describe the successful construction of four new drum-like Cd–Tb nanoclusters **1–4** from specifically designed Schiff base ligands H_2_L^1–3^. Four 1,4-BDC bridging units are introduced into the structure of **4**, resulting in superior luminescence properties compared to those of the related Cd–Tb nanocluster **2** which does not contain these groups. The enclosed 1,4-BDC units may not only act as efficient sensitizers for the lanthanide luminescence, but may also protect the lanthanide centers from the solvent environment. Green fluorescence is observed in SGC cells and PANC cells treated with **4**, and the fluorescence intensity gradually increases in step with the increase of treatment time and concentration of **4**. ICP-MS analysis shows that the cellular uptakes of **4** in 1000 SGC and PANC cells after treatment for 3 hours are 0.0094 pmol and 0.015 pmol, respectively. To the best of our knowledge, this is the first report on the biological application of high-nuclearity d–4f nanoclusters as cellular probes. Further studies focused on the construction of bioactive high-nuclearity d–f nanoclusters bearing flexible chain-like ligands with various carbon backbones and excellent luminescence properties are in progress.

## Conflicts of interest

There are no conflicts to declare.

## Supplementary Material

Supplementary information

Crystal structure data

## References

[cit1] Teng X., Zhu Y.-Z., Wei W., Wang S.-C., Huang J.-F., Naccache R., Hu W.-B., Tok A. I. Y., Han Y., Zhang Q.-C., Fan Q.-L., Huang W., Capobianco J. A., Huang L. (2012). J. Am. Chem. Soc..

[cit2] (a) Haugland R. P., A Guide to Fluorescent Probes and Labelling Technologies, Molecular Probes, Eugene, Oregon, 10th edn, 2005.

[cit3] Coogan M. P., Fernández-Moreira V. (2014). Chem. Commun..

[cit4] Beeby A., Clarkson I. M., Dickins R. S., Faulkner S., Parker D., Royle L., de Sousa A. S., Gareth Williams J. A., Woods M. (1999). J. Chem. Soc., Perkin Trans. 2.

[cit5] Peng J.-B., Zhang Q.-C., Kong X.-J., Zheng Y.-Z., Ren Y.-P., Long L.-S., Huang R.-B., Zheng L.-S., Zheng Z. (2012). J. Am. Chem. Soc..

[cit6] Michalet X., Pinaud F. F., Bentolila L. A., Tsay J. M., Doose S., Li J. J., Sundaresan G., Wu A. M., Gambhir S. S., Weiss S. (2005). Science.

[cit7] Thielemann D. T., Wagner A. T., Rösch E., Kölmel D. K., Heck J. G., Rudat B., Neumaier M., Feldmann C., Schepers U., Bräse S., Roesky P. W. (2013). J. Am. Chem. Soc..

[cit8] Li X.-L., Shi X. X.-L., Zhang L.-Y., Wen H.-M., Chen Z.-N. (2007). Inorg. Chem..

[cit9] Wen H.-M., Yang Y., Zhou X.-S., Liu J.-Y., Zhang D.-B., Chen Z.-B., Wang J.-Y., Chen Z.-N., Tian Z.-Q. (2013). Chem. Sci..

[cit10] Xu H.-B., Wen H.-M., Chen Z.-H., Li J., Shi L.-X., Chen Z.-N. (2010). Dalton Trans..

[cit11] Piguet C., Bünzli J.-C. G., Bernardinelli G., Hopfgartner G., Petoud S., Schaad O. (1996). J. Am. Chem. Soc..

[cit12] Chi Y.-X., Niu S.-Y., Jin J., Wang R., Li Y. (2009). Dalton Trans..

[cit13] Xiang S.-C., Hu S.-M., Sheng T.-L., Fu R.-B., Wu X.-T., Zhang X.-D. (2007). J. Am. Chem. Soc..

[cit14] Mereacre V. M., Ako A. M., Clerac R., Wernsdorfer W., Filoti G., Bartolome J., Anson C. E., Powell A. K. (2007). J. Am. Chem. Soc..

[cit15] Kong X.-J., Ren Y.-P., Long L.-S., Zheng Z., Huang R.-B., Zheng L.-S. (2007). J. Am. Chem. Soc..

[cit16] Sinha S., Gaur P., Dev S., Mukherjee T., Mathew J., Mukhopadhyay S., Ghosh S. (2015). Dalton Trans..

[cit17] Sakamoto M., Manseki K., Okawa H. (2001). Coord. Chem. Rev..

[cit18] Yang X.-P., Schipper D., Jones R. A., Lytwak L. A., Holliday B. J., Huang S.-M. (2013). J. Am. Chem. Soc..

[cit19] Yang X.-P., Li Z.-P., Wang S.-Q., Huang S.-M., Schipper D., Jones R. A. (2014). Chem. Commun..

[cit20] Cheng J.-W., Zhang J., Zheng S.-T., Zhang M.-B., Yang G.-Y. (2006). Angew. Chem., Int. Ed..

[cit21] Choppin G. R., Peterman D. R. (1998). Coord. Chem. Rev..

[cit22] Kreno L. E., Leong K., Farha O. K., Allendorf M., Van Duyne R. P., Hupp J. T. (2012). Chem. Rev..

[cit23] Lam F., Xu J.-X., Chan K.-S. (1996). J. Org. Chem..

[cit24] Fluorescence quantum yields were determined by using quinine sulfate (*Φ*_em_ = 0.546 in 0.5 M H_2_SO_4_) as a standard for the Cd–Tb nanoclusters: Meech S. R. Philips D. J., J. Photochem., 1983, 23 , 193 –217 .

[cit25] Otwinowski Z. and Minor W., Methods in Enzymology, 276: Macromolecular Crystallography, Part A, ed. C. W. J. Carter, M. I. Simon and R. M. Sweet, Academic Press, DENZO-SMN, 1997, pp. 307–326. 10.1016/S0076-6879(97)76066-X 27754618

[cit26] Sheldrick G. H., SHELX 97, A software package for the solution and refinement of X-ray data, University of Göttingen, Göttingen, Germany, 1997.

[cit27] Cromer D. T. and Waber J. T., International Tables for X-Ray Crystallography, Kynoch Press, Birmingham, 1974, vol. 4, Table 2.2A.

