# Supplementary material for: A self-assembling luminescent lanthanide molecular nanoparticle with potential for live cell imaging
Source: Chem Sci. 2018 Apr 26;9(20):4630–7. doi: 10.1039/c8sc00650d (PMC5969494; doi:10.1039/c8sc00650d)
Supplement: Supplementary file 1 [file SC-009-C8SC00650D-s001.pdf]

Electronic Supplementary Material (ESI) for Chemical Science.
This journal is © The Royal Society of Chemistry 2018

Supporting Information

A self-assembling lanthanide molecular nanoparticle with enhanced luminescence for live cell imaging

Xiaoping Yang, Shiqing Wang, Yali Zhang, Guang Liang, Ting Zhu, Lijie Zhang, Shaoming Huang, Desmond Schipper and Richard A. Jones

Contents

1. Powder XRD patterns of Cd-Tb nanoclusters **1-4**…………………………..………………..S2
2. Excitation spectra for **1-4** ………..…………………..…...S4
3. Cytotoxicity assays ………..…………………..…...S5
4. Cell imaging study ………..…………………..…....S6
5. ICP-MS analysis for cellular uptake...…………….…..…....S7
6. X-Ray Crystallography……………..………………………………....…...…...……......…S8

1. Powder XRD patterns of Cd-Tb nanoclusters 1-4

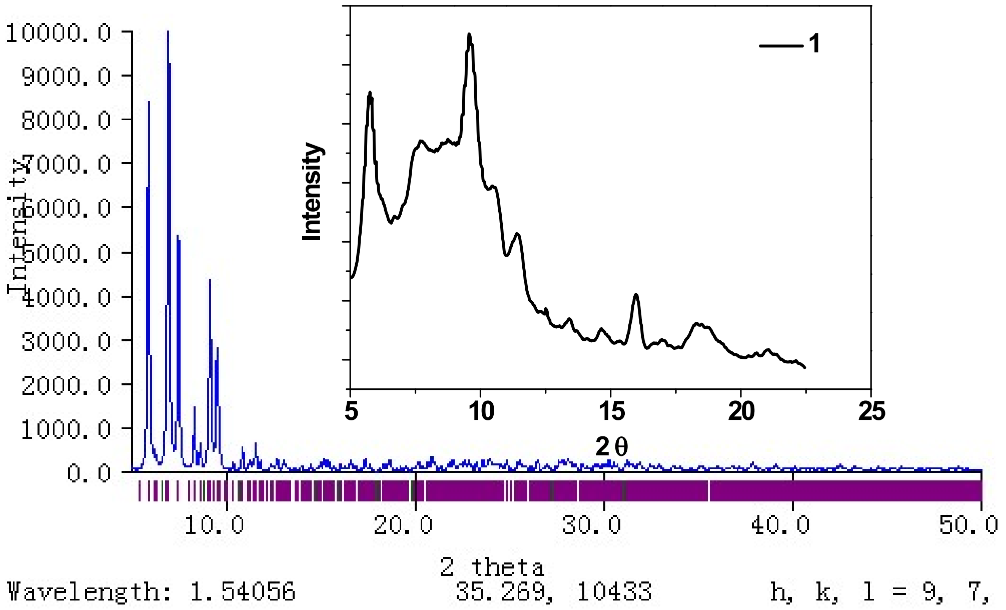

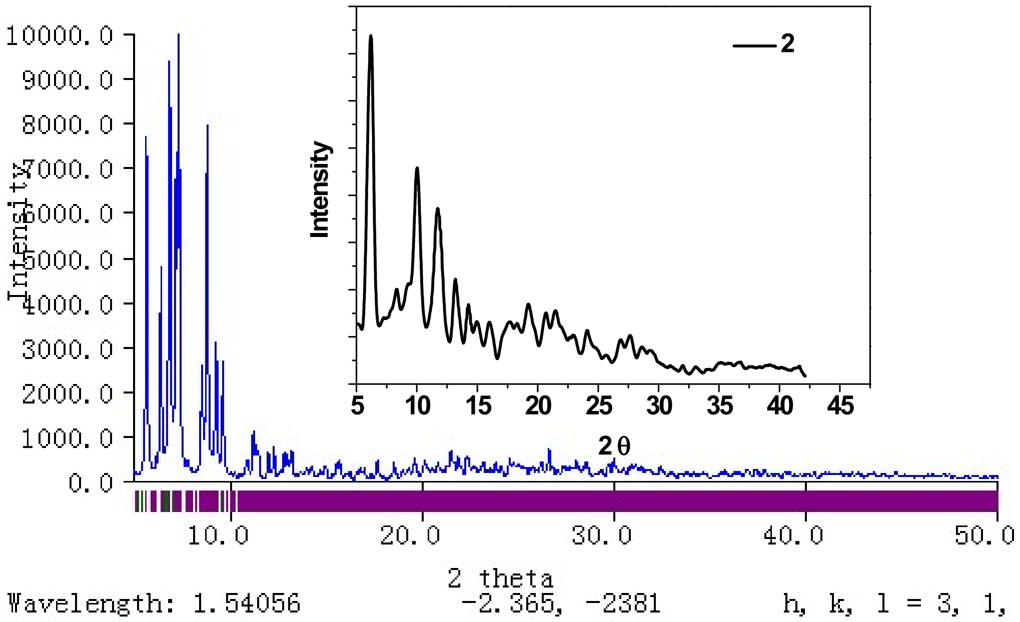

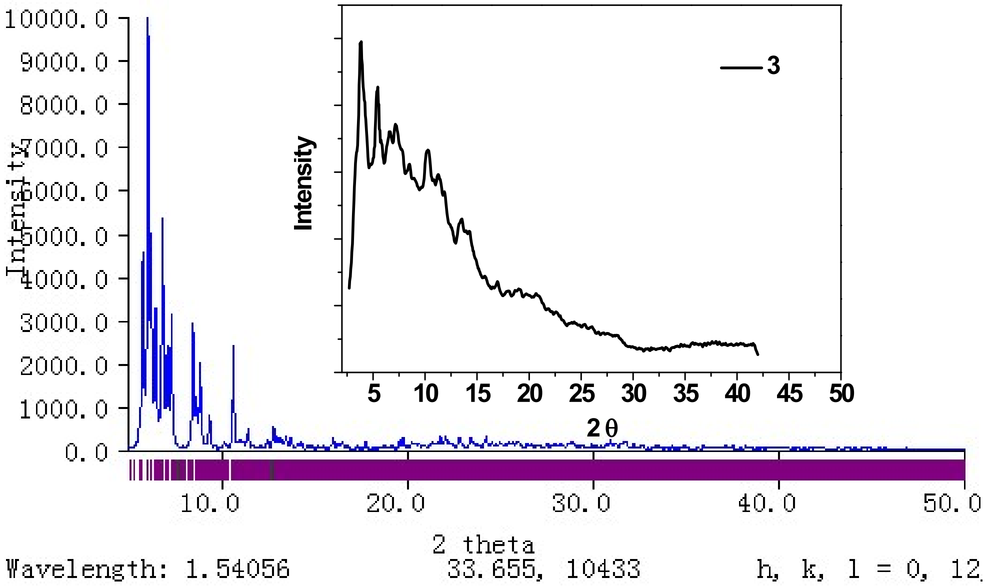

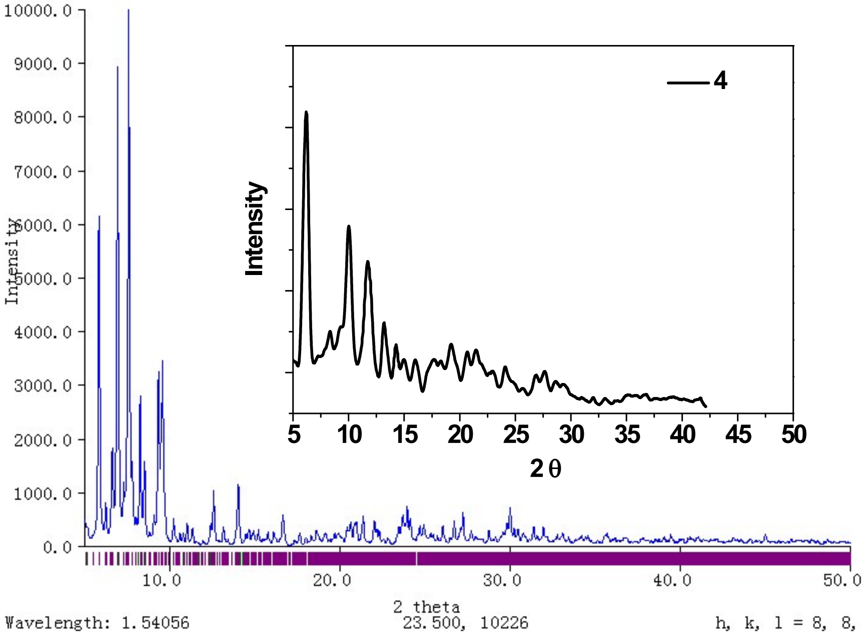

Figure S1. Powder XRD patterns of **1**-**4**

2. Excitation spectra for 1-4

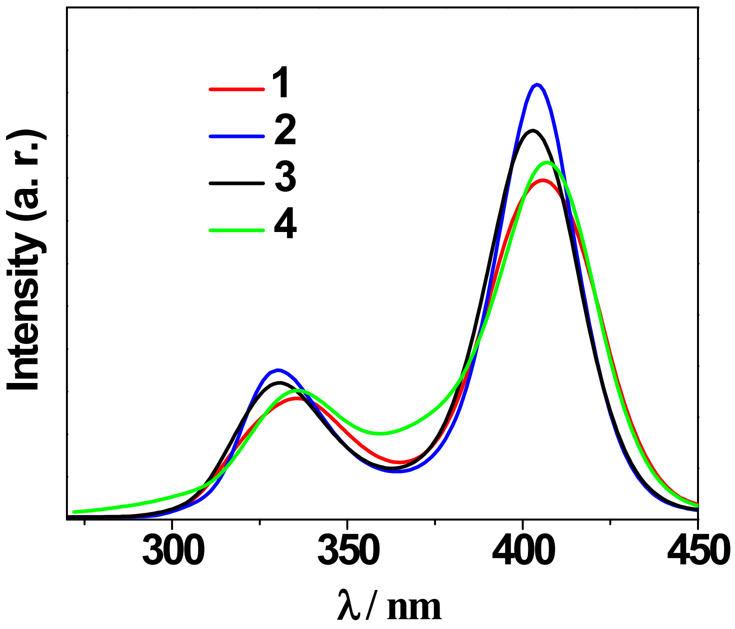

Figure S2. Excitation spectra for **1-4**.

3. Cytotoxicity assays

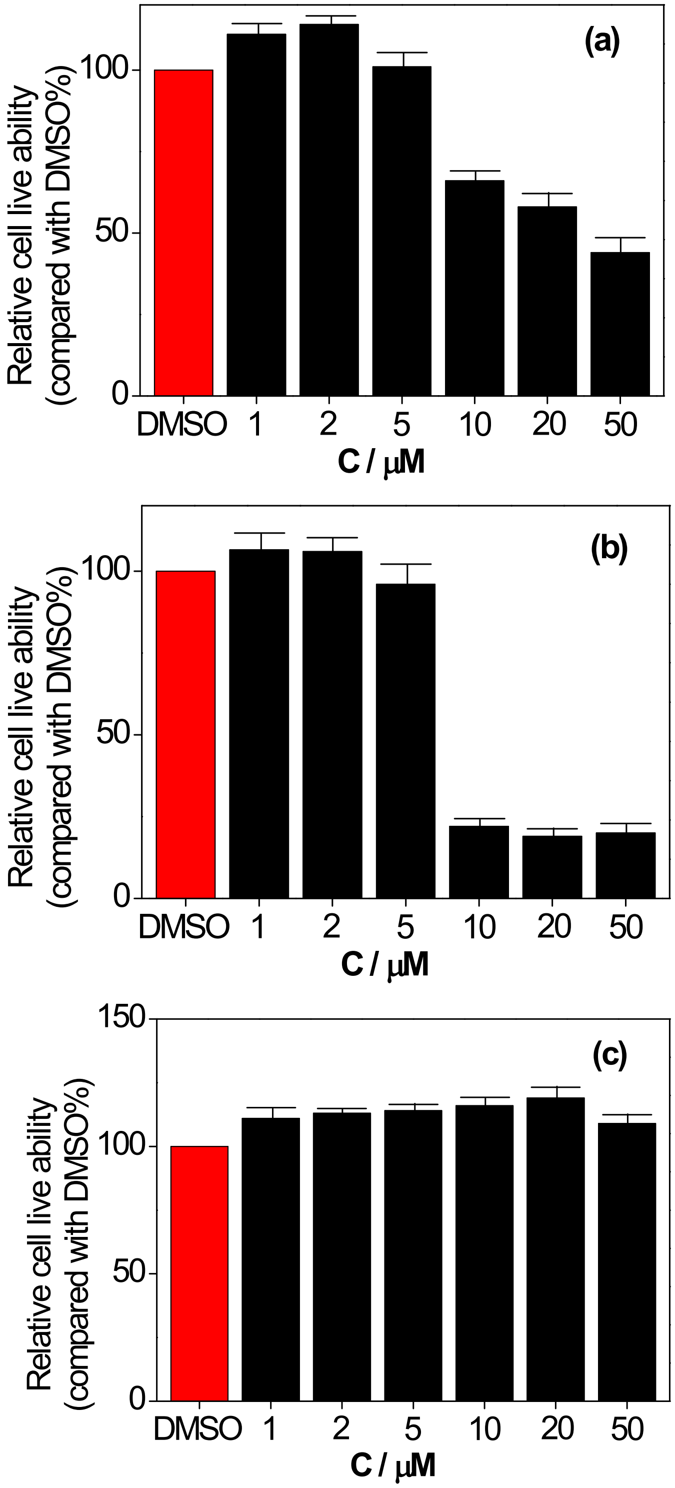

Figure S3. Dose responsive cell proliferation curves of SGC cells treated with H_2L^2 (a), $Cd(OAc)_2 \cdot 2H_2O$ (b) and $Tb(OAc)_3 \cdot 4H_2O$ (c).

4. Cell imaging study

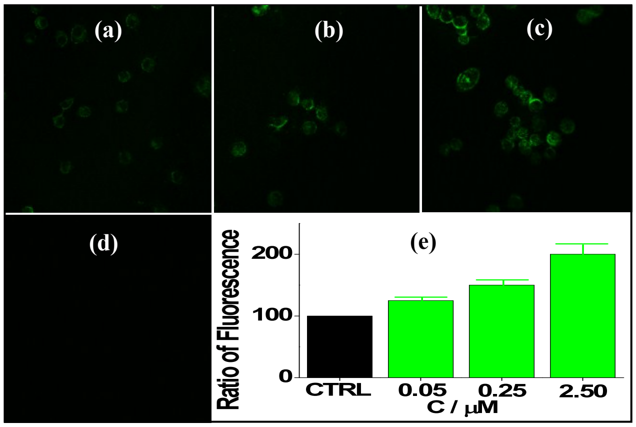

Figure S4. Fluorescence microscopic photographs of SGC cells treated with 4 for 30 minutes (concentrations of **4**: 0 μM (a), 0.05 μM (b), 0.25 μM (c), and 2.50 μM (d)).

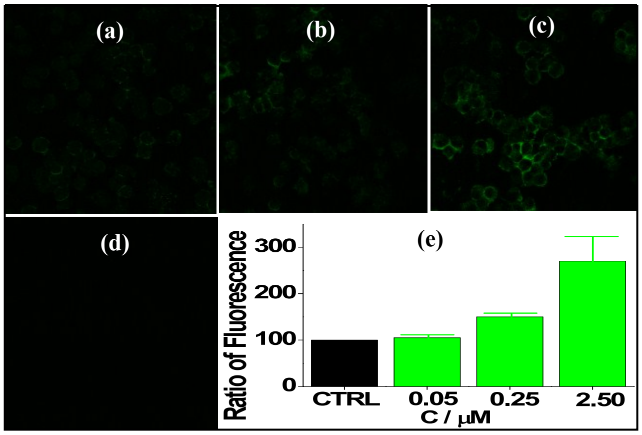

Figure S5. Fluorescence microscopic photographs of PANC cells treated with **4** for 30 minutes (concentrations of **4**: 0 nM (a), 1 nM (b), 5 nM (c), and 50 nM (d)).

5. ICP-MS analysis for cellular uptake

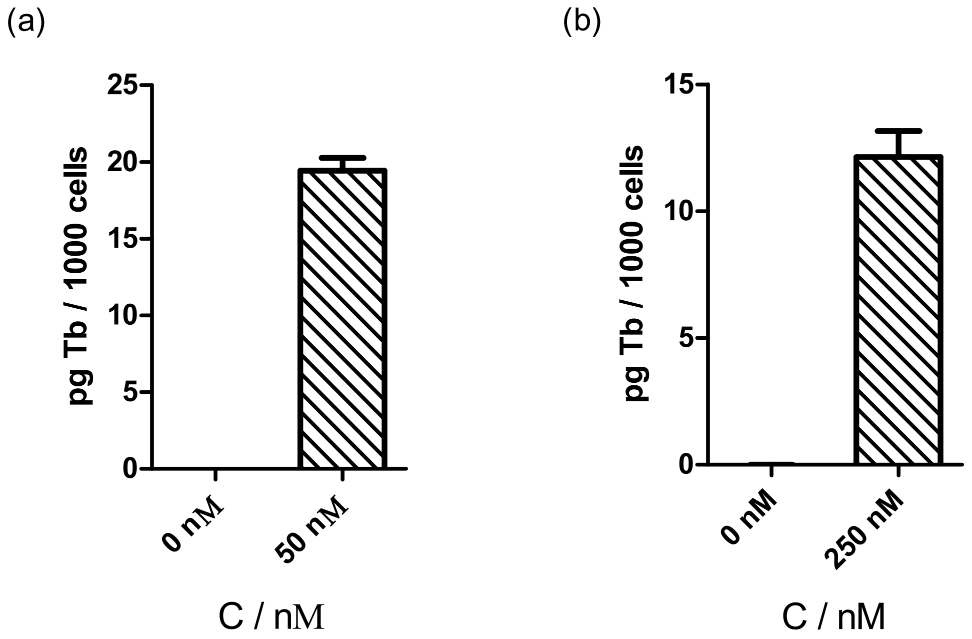

Figure S6. (a) Cellular Tb(III) concentration in PANC cells after 3 hours exposure to **4** (50 nM). (b) Cellular Tb(III) concentration in SGC cells after 3 hours exposure to **4** (250 nM).

6. X-Ray Crystallography

Table S1. Selected Bond Lengths (Å) and Angles (°) for **1**.

Tb(1)-O(10)	2.344(9)
Tb(1)-O(21)	2.349(10)
Tb(1)-O(24)	2.349(9)
Tb(1)-O(6)	2.395(10)
Tb(1)-O(20)	2.397(9)
Tb(1)-O(18)	2.405(10)
Tb(1)-N(3)	2.599(12)
Tb(1)-O(9)	2.614(10)
Tb(2)-O(26)	2.289(9)
Tb(2)-O(7)	2.303(8)
Tb(2)-O(3)	2.326(8)
Tb(2)-O(28)	2.346(9)
Tb(2)-O(29)	2.406(8)
Tb(2)-O(31)	2.416(9)
Tb(2)-N(2)	2.614(10)
Tb(2)-O(8)	2.618(8)
Cd(1)-O(2)	2.249(10)
Cd(1)-N(1)	2.252(12)
Cd(1)-O(17)	2.254(10)
Cd(1)-O(6)	2.267(10)
Cd(1)-O(20)	2.343(9)
Cd(1)-O(5)	2.551(10)
Cd(1)-O(19)	2.584(9)
Cd(2)-O(22)	2.250(10)
Cd(2)-O(23)	2.251(9)
Cd(2)-O(10)	2.271(9)
Cd(2)-N(5)#1	2.332(11)
Cd(2)-O(14)#1	2.355(10)
Cd(2)-O(19)#1	2.376(9)
Cd(3)-O(27)	2.228(8)
Cd(3)-O(7)	2.302(8)
Cd(3)-N(4)	2.306(10)
Cd(3)-O(25)	2.320(8)
Cd(3)-O(30)#1	2.359(8)
Cd(3)-O(36)	2.432(9)
Cd(4)-O(11)	2.233(8)
Cd(4)-N(6)	2.234(11)
Cd(4)-O(32)	2.260(8)
Cd(4)-O(3)	2.281(8)
Cd(4)-O(29)	2.308(8)
Cd(4)-O(4)	2.521(8)
Cd(5)-O(11)	2.261(8)
Cd(5)-O(32)	2.269(8)
Cd(5)-O(34)	2.278(9)
Cd(5)-O(35)	2.305(9)
Cd(5)-O(36)#2	2.402(8)
Cd(5)-O(12)	2.459(8)
Cd(5)-O(33)	2.521(10)
Cd(6)-O(2)	2.236(11)
Cd(6)-O(15)	2.264(13)
Cd(6)-O(17)	2.293(10)
Cd(6)-O(13)	2.294(13)
Cd(6)-O(1)	2.427(12)
Cd(6)-O(14)	2.441(10)
Cd(6)-O(16)	2.588(16)
O(10)-Tb(1)-O(21)	79.1(3)
O(10)-Tb(1)-O(24)	83.3(3)
O(21)-Tb(1)-O(24)	75.7(3)
O(10)-Tb(1)-O(6)	129.7(3)
O(21)-Tb(1)-O(6)	146.2(3)
O(24)-Tb(1)-O(6)	119.5(3)
O(10)-Tb(1)-O(20)	159.5(3)
O(21)-Tb(1)-O(20)	85.4(3)
O(24)-Tb(1)-O(20)	79.9(3)
O(6)-Tb(1)-O(20)	69.7(3)
O(10)-Tb(1)-O(18)	110.1(3)
O(21)-Tb(1)-O(18)	72.3(4)
O(24)-Tb(1)-O(18)	141.8(3)
O(6)-Tb(1)-O(18)	80.0(3)
O(20)-Tb(1)-O(18)	77.3(3)
O(10)-Tb(1)-N(3)	73.5(3)
O(21)-Tb(1)-N(3)	144.4(4)
O(24)-Tb(1)-N(3)	78.8(3)
O(6)-Tb(1)-N(3)	69.0(4)
O(20)-Tb(1)-N(3)	114.2(3)
O(18)-Tb(1)-N(3)	138.8(4)
O(10)-Tb(1)-O(9)	62.7(3)
O(21)-Tb(1)-O(9)	111.6(3)
O(24)-Tb(1)-O(9)	141.8(3)
O(6)-Tb(1)-O(9)	76.1(3)
O(20)-Tb(1)-O(9)	136.6(3)
O(18)-Tb(1)-O(9)	71.2(3)
N(3)-Tb(1)-O(9)	75.3(4)
O(26)-Tb(2)-O(7)	86.0(3)
O(26)-Tb(2)-O(3)	115.2(3)
O(7)-Tb(2)-O(3)	128.6(3)
O(26)-Tb(2)-O(28)	75.7(3)
O(7)-Tb(2)-O(28)	78.2(3)
O(3)-Tb(2)-O(28)	149.8(3)
O(26)-Tb(2)-O(29)	78.7(3)
O(7)-Tb(2)-O(29)	160.5(3)
O(3)-Tb(2)-O(29)	69.7(3)
O(28)-Tb(2)-O(29)	86.2(3)
O(26)-Tb(2)-O(31)	142.1(3)
O(7)-Tb(2)-O(31)	109.1(3)
O(3)-Tb(2)-O(31)	82.9(3)

O(28)-Tb(2)-O(31)	73.9(3)	O(7)-Tb(2)-N(2)	72.5(3)
O(29)-Tb(2)-O(31)	77.1(3)	O(3)-Tb(2)-N(2)	69.2(3)
O(26)-Tb(2)-N(2)	75.7(3)	O(28)-Tb(2)-N(2)	140.0(3)
O(29)-Tb(2)-N(2)	114.7(3)	O(22)-Cd(2)-O(23)	91.5(3)
O(31)-Tb(2)-N(2)	141.5(3)	O(22)-Cd(2)-O(10)	109.8(3)
O(26)-Tb(2)-O(8)	144.1(3)	O(23)-Cd(2)-O(10)	93.0(3)
O(7)-Tb(2)-O(8)	62.2(3)	O(22)-Cd(2)-N(5)#1	172.4(4)
O(3)-Tb(2)-O(8)	77.8(3)	O(23)-Cd(2)-N(5)#1	90.4(4)
O(28)-Tb(2)-O(8)	110.7(3)	O(10)-Cd(2)-N(5)#1	77.5(4)
O(29)-Tb(2)-O(8)	135.7(3)	O(22)-Cd(2)-O(14)#1	82.9(4)
O(31)-Tb(2)-O(8)	69.5(3)	O(23)-Cd(2)-O(14)#1	174.0(4)
N(2)-Tb(2)-O(8)	78.9(3)	O(10)-Cd(2)-O(14)#1	86.8(3)
O(2)-Cd(1)-N(1)	82.9(4)	N(5)#1-Cd(2)-O(14)#1	95.4(4)
O(2)-Cd(1)-O(17)	76.1(4)	O(22)-Cd(2)-O(19)#1	87.1(3)
N(1)-Cd(1)-O(17)	158.9(4)	O(23)-Cd(2)-O(19)#1	92.4(3)
O(2)-Cd(1)-O(6)	147.4(3)	O(10)-Cd(2)-O(19)#1	162.1(3)
N(1)-Cd(1)-O(6)	100.0(4)	N(5)#1-Cd(2)-O(19)#1	85.4(4)
O(17)-Cd(1)-O(6)	96.8(4)	O(14)#1-Cd(2)-O(19)#1	89.6(3)
O(2)-Cd(1)-O(20)	136.5(3)	O(27)-Cd(3)-O(7)	107.1(3)
N(1)-Cd(1)-O(20)	111.4(4)	O(27)-Cd(3)-N(4)	168.8(3)
O(17)-Cd(1)-O(20)	85.7(3)	O(7)-Cd(3)-N(4)	79.3(3)
O(6)-Cd(1)-O(20)	72.8(3)	O(27)-Cd(3)-O(25)	92.3(3)
O(2)-Cd(1)-O(5)	82.0(4)	O(7)-Cd(3)-O(25)	96.3(3)
N(1)-Cd(1)-O(5)	87.4(4)	N(4)-Cd(3)-O(25)	96.1(3)
O(17)-Cd(1)-O(5)	87.9(4)	O(27)-Cd(3)-O(30)#1	87.4(3)
O(6)-Cd(1)-O(5)	65.9(3)	O(7)-Cd(3)-O(30)#1	162.1(3)
O(20)-Cd(1)-O(5)	137.1(3)	N(4)-Cd(3)-O(30)#1	84.7(3)
O(2)-Cd(1)-O(19)	86.8(3)	O(25)-Cd(3)-O(30)#1	93.5(3)
N(1)-Cd(1)-O(19)	99.9(4)	O(27)-Cd(3)-O(36)	81.8(3)
O(17)-Cd(1)-O(19)	80.9(3)	O(7)-Cd(3)-O(36)	83.5(3)
O(6)-Cd(1)-O(19)	123.9(3)	N(4)-Cd(3)-O(36)	90.1(3)
O(20)-Cd(1)-O(19)	51.1(3)	O(25)-Cd(3)-O(36)	173.6(3)
O(5)-Cd(1)-O(19)	165.8(3)	O(30)#1-Cd(3)-O(36)	88.4(3)

Table S2. Selected Bond Lengths (Å) and Angles (°) for **2**.

Tb(1)-O(67)	2.353(15)	Cd(6)-O(1)	2.550(15)
Tb(1)-O(65)	2.366(15)	O(67)-Tb(1)-O(65)	73.3(5)
Tb(1)-O(19)	2.388(13)	O(67)-Tb(1)-O(19)	119.5(5)
Tb(1)-O(15)	2.409(15)	O(65)-Tb(1)-O(19)	150.6(5)
Tb(1)-O(68)	2.477(16)	O(67)-Tb(1)-O(15)	85.7(5)
Tb(1)-O(50)#1	2.499(15)	O(65)-Tb(1)-O(15)	79.0(5)
Tb(1)-N(10)	2.593(16)	O(19)-Tb(1)-O(15)	125.8(4)
Tb(1)-O(16)	2.653(14)	O(67)-Tb(1)-O(68)	140.7(5)
Tb(2)-O(46)	2.369(16)	O(65)-Tb(1)-O(68)	74.1(5)
Tb(2)-O(44)	2.392(19)	O(19)-Tb(1)-O(68)	82.0(5)
Tb(2)-O(18)	2.399(14)	O(15)-Tb(1)-O(68)	108.9(5)
Tb(2)-O(14)	2.406(15)	O(67)-Tb(1)-O(50)#1	82.4(5)
Tb(2)-O(43)	2.436(16)	O(65)-Tb(1)-O(50)#1	89.0(5)
Tb(2)-O(41)	2.475(15)	O(19)-Tb(1)-O(50)#1	68.4(4)
Tb(2)-O(17)	2.645(15)	O(15)-Tb(1)-O(50)#1	165.1(5)
Tb(2)-N(7)	2.661(19)	O(68)-Tb(1)-O(50)#1	75.7(5)
Cd(1)-O(35)	2.253(16)	O(67)-Tb(1)-N(10)	76.0(5)
Cd(1)-O(33)	2.313(18)	O(65)-Tb(1)-N(10)	138.1(5)
Cd(1)-N(3)	2.31(2)	O(19)-Tb(1)-N(10)	70.6(5)
Cd(1)-O(6)	2.325(16)	O(15)-Tb(1)-N(10)	70.9(5)
Cd(1)-O(40)	2.351(16)	O(68)-Tb(1)-N(10)	142.9(5)
Cd(1)-O(36)	2.415(15)	O(50)#1-Tb(1)-N(10)	114.4(5)
Cd(2)-O(54)	2.234(16)	O(67)-Tb(1)-O(16)	142.5(5)
Cd(2)-O(52)	2.304(17)	O(65)-Tb(1)-O(16)	111.6(5)
Cd(2)-N(2)	2.330(17)	O(19)-Tb(1)-O(16)	75.3(5)
Cd(2)-O(3)	2.342(15)	O(15)-Tb(1)-O(16)	60.4(5)
Cd(2)-O(49)	2.376(14)	O(68)-Tb(1)-O(16)	71.2(5)
Cd(2)-O(51)	2.416(14)	O(50)#1-Tb(1)-O(16)	133.4(4)
Cd(3)-O(45)	2.248(18)	N(10)-Tb(1)-O(16)	77.9(5)
Cd(3)-O(18)	2.318(15)	O(46)-Tb(2)-O(44)	76.4(6)
Cd(3)-O(47)	2.325(16)	O(46)-Tb(2)-O(18)	84.8(5)
Cd(3)-N(9)	2.336(18)	O(44)-Tb(2)-O(18)	76.9(5)
Cd(3)-O(30)#1	2.355(15)	O(46)-Tb(2)-O(14)	116.9(5)
Cd(3)-O(48)	2.393(17)	O(44)-Tb(2)-O(14)	150.5(5)
Cd(4)-O(11)	2.226(15)	O(18)-Tb(2)-O(14)	128.1(5)
Cd(4)-N(6)	2.264(19)	O(46)-Tb(2)-O(43)	142.3(6)
Cd(4)-O(7)	2.288(15)	O(44)-Tb(2)-O(43)	74.3(6)
Cd(4)-O(57)	2.291(16)	O(18)-Tb(2)-O(43)	110.7(5)
Cd(4)-O(58)	2.312(15)	O(14)-Tb(2)-O(43)	81.2(5)
Cd(4)-O(8)	2.524(17)	O(46)-Tb(2)-O(41)	80.1(5)
Cd(5)-O(37)	2.264(16)	O(44)-Tb(2)-O(41)	88.2(6)
Cd(5)-O(10)	2.266(15)	O(18)-Tb(2)-O(41)	160.9(5)
Cd(5)-O(42)	2.324(14)	O(14)-Tb(2)-O(41)	69.9(5)
Cd(5)-O(38)	2.335(16)	O(43)-Tb(2)-O(41)	75.7(5)
Cd(5)-O(9)	2.462(16)	O(46)-Tb(2)-O(17)	142.5(5)
Cd(5)-O(36)	2.470(15)	O(44)-Tb(2)-O(17)	107.6(6)
Cd(5)-O(39)	2.535(17)	O(18)-Tb(2)-O(17)	61.1(5)
Cd(6)-O(22)#1	2.219(16)	O(14)-Tb(2)-O(17)	78.4(5)
Cd(6)-N(11)#1	2.272(19)	O(43)-Tb(2)-O(17)	70.3(5)
Cd(6)-O(31)	2.310(14)	O(41)-Tb(2)-O(17)	136.3(5)
Cd(6)-O(2)	2.319(17)	O(46)-Tb(2)-N(7)	77.3(6)
Cd(6)-O(28)	2.322(16)	O(44)-Tb(2)-N(7)	140.5(6)

O(18)-Tb(2)-N(7)	71.8(5)
O(14)-Tb(2)-N(7)	68.7(5)
O(43)-Tb(2)-N(7)	139.6(6)
O(41)-Tb(2)-N(7)	115.6(5)
O(17)-Tb(2)-N(7)	77.5(5)
O(35)-Cd(1)-O(33)	91.6(6)
O(35)-Cd(1)-N(3)	169.1(7)
O(33)-Cd(1)-N(3)	96.6(7)
O(35)-Cd(1)-O(6)	107.0(6)
O(33)-Cd(1)-O(6)	97.0(6)
N(3)-Cd(1)-O(6)	79.2(6)
O(35)-Cd(1)-O(40)	87.2(6)
O(33)-Cd(1)-O(40)	94.0(6)
N(3)-Cd(1)-O(40)	85.0(6)
O(6)-Cd(1)-O(40)	161.7(6)
O(35)-Cd(1)-O(36)	83.5(6)
O(33)-Cd(1)-O(36)	174.7(6)
N(3)-Cd(1)-O(36)	88.5(6)
O(6)-Cd(1)-O(36)	82.8(5)
O(40)-Cd(1)-O(36)	87.6(5)
O(54)-Cd(2)-O(52)	91.0(6)
O(54)-Cd(2)-N(2)	167.5(6)
O(52)-Cd(2)-N(2)	97.0(6)
O(54)-Cd(2)-O(3)	111.4(6)
O(52)-Cd(2)-O(3)	95.8(6)
N(2)-Cd(2)-O(3)	77.5(6)
O(54)-Cd(2)-O(49)	84.8(5)
O(52)-Cd(2)-O(49)	93.2(5)
N(2)-Cd(2)-O(49)	85.2(6)
O(3)-Cd(2)-O(49)	161.4(5)
O(54)-Cd(2)-O(51)	83.8(6)
O(52)-Cd(2)-O(51)	174.7(5)
N(2)-Cd(2)-O(51)	88.3(6)
O(3)-Cd(2)-O(51)	84.9(5)
O(49)-Cd(2)-O(51)	87.7(5)
O(45)-Cd(3)-O(18)	104.9(6)
O(45)-Cd(3)-O(47)	93.6(6)
O(18)-Cd(3)-O(47)	98.5(5)
O(45)-Cd(3)-N(9)	168.5(6)
O(18)-Cd(3)-N(9)	78.0(6)
O(47)-Cd(3)-N(9)	97.1(6)
O(45)-Cd(3)-O(30)#1	89.0(6)
O(18)-Cd(3)-O(30)#1	162.9(5)
O(47)-Cd(3)-O(30)#1	90.5(5)
N(9)-Cd(3)-O(30)#1	86.5(6)
O(45)-Cd(3)-O(48)	83.3(6)
O(18)-Cd(3)-O(48)	85.7(5)
O(47)-Cd(3)-O(48)	175.2(6)
N(9)-Cd(3)-O(48)	85.8(6)
O(30)#1-Cd(3)-O(48)	85.9(5)
O(11)-Cd(4)-N(6)	83.8(6)
O(11)-Cd(4)-O(7)	150.2(6)
N(6)-Cd(4)-O(7)	98.5(6)
O(11)-Cd(4)-O(57)	75.5(5)
N(6)-Cd(4)-O(57)	159.3(6)
O(7)-Cd(4)-O(57)	99.0(5)
O(11)-Cd(4)-O(58)	133.4(6)
N(6)-Cd(4)-O(58)	107.1(6)
O(7)-Cd(4)-O(58)	74.4(6)
O(57)-Cd(4)-O(58)	88.2(5)
O(11)-Cd(4)-O(8)	85.1(5)
N(6)-Cd(4)-O(8)	85.9(6)
O(7)-Cd(4)-O(8)	65.6(6)
O(57)-Cd(4)-O(8)	91.4(6)
O(58)-Cd(4)-O(8)	139.5(5)
O(37)-Cd(5)-O(10)	144.5(6)
O(37)-Cd(5)-O(42)	112.3(6)
O(10)-Cd(5)-O(42)	75.9(5)
O(37)-Cd(5)-O(38)	104.1(6)
O(10)-Cd(5)-O(38)	101.2(6)
O(42)-Cd(5)-O(38)	117.9(5)
O(37)-Cd(5)-O(9)	95.6(6)
O(10)-Cd(5)-O(9)	66.6(5)
O(42)-Cd(5)-O(9)	142.0(6)
O(38)-Cd(5)-O(9)	76.7(6)
O(37)-Cd(5)-O(36)	54.2(5)
O(10)-Cd(5)-O(36)	93.9(5)
O(42)-Cd(5)-O(36)	86.2(5)
O(38)-Cd(5)-O(36)	153.9(5)
O(9)-Cd(5)-O(36)	90.2(5)
O(37)-Cd(5)-O(39)	86.9(6)
O(10)-Cd(5)-O(39)	128.3(6)
O(42)-Cd(5)-O(39)	78.7(6)
O(38)-Cd(5)-O(39)	54.6(6)
O(9)-Cd(5)-O(39)	130.1(6)
O(36)-Cd(5)-O(39)	128.5(6)
O(22)#1-Cd(6)-N(11)#1	83.6(6)
O(22)#1-Cd(6)-O(31)	134.4(5)
N(11)#1-Cd(6)-O(31)	107.6(6)
O(22)#1-Cd(6)-O(2)	149.7(6)
N(11)#1-Cd(6)-O(2)	96.0(6)
O(31)-Cd(6)-O(2)	74.6(6)
O(22)#1-Cd(6)-O(28)	75.6(6)
N(11)#1-Cd(6)-O(28)	159.1(6)
O(31)-Cd(6)-O(28)	88.9(5)
O(2)-Cd(6)-O(28)	100.8(6)
O(22)#1-Cd(6)-O(1)	83.6(5)
N(11)#1-Cd(6)-O(1)	87.7(6)
O(31)-Cd(6)-O(1)	139.2(6)
O(2)-Cd(6)-O(1)	66.2(6)
O(28)-Cd(6)-O(1)	87.9(5)

Table S3. Selected Bond Lengths (Å) and Angles (°) for **3**.

Tb(1)-O(50)	2.277(12)
Tb(1)-O(23)#1	2.295(12)
Tb(1)-O(52)	2.299(14)
Tb(1)-O(19)#1	2.316(11)
Tb(1)-O(53)	2.331(11)
Tb(1)-O(56)	2.349(12)
Tb(1)-N(12)#1	2.553(15)
Tb(1)-O(20)#1	2.570(11)
Tb(2)-O(45)	2.289(11)
Tb(2)-O(22)	2.291(12)
Tb(2)-O(43)	2.316(11)
Tb(2)-O(18)	2.339(11)
Tb(2)-O(42)	2.344(12)
Tb(2)-O(38)	2.353(12)
Tb(2)-N(9)	2.531(14)
Tb(2)-O(21)	2.596(11)
Cd(4)-O(31)	2.268(12)
Cd(4)-O(33)	2.296(12)
Cd(4)-O(10)	2.297(12)
Cd(4)-N(5)	2.350(16)
Cd(4)-O(37)	2.351(12)
Cd(4)-O(36)	2.391(12)
Cd(5)-O(2)	2.215(12)
Cd(5)-O(27)	2.267(12)
Cd(5)-N(1)	2.268(16)
Cd(5)-O(30)	2.319(12)
Cd(5)-O(6)	2.326(12)
Cd(5)-O(5)	2.535(16)
Cd(5)-O(29)	2.625(12)
Cd(6)-O(14)	2.254(12)
Cd(6)-O(41)	2.311(12)
Cd(6)-O(35)	2.321(12)
Cd(6)-O(40)	2.333(13)
Cd(6)-O(13)	2.416(13)
Cd(6)-O(36)	2.417(11)
Cd(6)-O(39)	2.446(14)
Cd(8)-O(44)	2.261(12)
Cd(8)-O(22)	2.283(11)
Cd(8)-O(46)	2.296(13)
Cd(8)-N(11)	2.331(15)
Cd(8)-O(29)#1	2.361(12)
Cd(8)-O(47)	2.398(12)
O(50)-Tb(1)-O(23)#1	116.2(4)
O(50)-Tb(1)-O(52)	76.3(4)
O(23)#1-Tb(1)-O(52)	149.6(4)
O(50)-Tb(1)-O(19)#1	84.9(4)
O(23)#1-Tb(1)-O(19)#1	128.4(4)
O(52)-Tb(1)-O(19)#1	78.2(4)
O(50)-Tb(1)-O(53)	141.3(4)
O(23)#1-Tb(1)-O(53)	82.1(4)
O(52)-Tb(1)-O(53)	73.2(4)
O(19)#1-Tb(1)-O(53)	111.1(4)
O(50)-Tb(1)-O(56)	78.1(4)
O(23)#1-Tb(1)-O(56)	69.9(4)
O(52)-Tb(1)-O(56)	87.3(4)
O(19)#1-Tb(1)-O(56)	159.8(4)
O(53)-Tb(1)-O(56)	77.2(4)
O(50)-Tb(1)-N(12)#1	76.6(5)
O(23)#1-Tb(1)-N(12)#1	68.9(4)
O(52)-Tb(1)-N(12)#1	141.0(4)
O(19)#1-Tb(1)-N(12)#1	71.9(4)
O(53)-Tb(1)-N(12)#1	141.1(5)
O(56)-Tb(1)-N(12)#1	113.8(4)
O(50)-Tb(1)-O(20)#1	144.9(4)
O(23)#1-Tb(1)-O(20)#1	76.7(4)
O(52)-Tb(1)-O(20)#1	109.7(4)
O(19)#1-Tb(1)-O(20)#1	63.7(4)
O(53)-Tb(1)-O(20)#1	69.6(4)
O(56)-Tb(1)-O(20)#1	135.4(4)
N(12)#1-Tb(1)-O(20)#1	78.8(4)
O(45)-Tb(2)-O(22)	86.2(4)
O(45)-Tb(2)-O(43)	75.2(4)
O(22)-Tb(2)-O(43)	79.0(4)
O(45)-Tb(2)-O(18)	117.9(4)
O(22)-Tb(2)-O(18)	128.4(4)
O(43)-Tb(2)-O(18)	147.9(4)
O(45)-Tb(2)-O(42)	140.4(4)
O(22)-Tb(2)-O(42)	108.6(4)
O(43)-Tb(2)-O(42)	72.3(4)
O(18)-Tb(2)-O(42)	81.8(4)
O(45)-Tb(2)-O(38)	78.2(4)
O(22)-Tb(2)-O(38)	160.8(4)
O(43)-Tb(2)-O(38)	86.2(4)
O(18)-Tb(2)-O(38)	69.7(4)
O(42)-Tb(2)-O(38)	77.8(4)
O(45)-Tb(2)-N(9)	75.8(4)
O(22)-Tb(2)-N(9)	73.1(4)
O(43)-Tb(2)-N(9)	140.6(5)
O(18)-Tb(2)-N(9)	70.6(4)
O(42)-Tb(2)-N(9)	143.1(4)
O(38)-Tb(2)-N(9)	113.0(4)
O(45)-Tb(2)-O(21)	144.8(4)
O(22)-Tb(2)-O(21)	62.8(4)
O(43)-Tb(2)-O(21)	111.9(4)
O(18)-Tb(2)-O(21)	75.3(4)
O(42)-Tb(2)-O(21)	70.2(4)
O(38)-Tb(2)-O(21)	135.2(4)
N(9)-Tb(2)-O(21)	79.2(4)
O(31)-Cd(4)-O(33)	90.1(4)
O(31)-Cd(4)-O(10)	110.9(4)
O(33)-Cd(4)-O(10)	92.3(4)
O(31)-Cd(4)-N(5)	168.3(5)
O(33)-Cd(4)-N(5)	94.8(5)
O(10)-Cd(4)-N(5)	79.5(5)

O(31)-Cd(4)-O(37)	86.6(4)	O(14)-Cd(6)-O(40)	97.8(4)
O(33)-Cd(4)-O(37)	93.5(4)	O(41)-Cd(6)-O(40)	111.6(4)
O(10)-Cd(4)-O(37)	161.5(4)	O(35)-Cd(6)-O(40)	109.2(4)
N(5)-Cd(4)-O(37)	82.6(5)	O(14)-Cd(6)-O(13)	67.3(4)
O(31)-Cd(4)-O(36)	84.5(4)	O(41)-Cd(6)-O(13)	141.1(4)
O(33)-Cd(4)-O(36)	173.9(4)	O(35)-Cd(6)-O(13)	89.6(4)
O(10)-Cd(4)-O(36)	87.1(4)	O(40)-Cd(6)-O(13)	79.6(5)
N(5)-Cd(4)-O(36)	91.0(5)	O(14)-Cd(6)-O(36)	91.4(4)
O(37)-Cd(4)-O(36)	89.0(4)	O(41)-Cd(6)-O(36)	85.9(4)
O(2)-Cd(5)-O(27)	75.8(5)	O(35)-Cd(6)-O(36)	55.7(4)
O(2)-Cd(5)-N(1)	83.4(5)	O(40)-Cd(6)-O(36)	161.8(4)
O(27)-Cd(5)-N(1)	158.9(5)	O(13)-Cd(6)-O(36)	89.7(4)
O(2)-Cd(5)-O(30)	138.5(5)	O(14)-Cd(6)-O(39)	132.3(5)
O(27)-Cd(5)-O(30)	88.3(5)	O(41)-Cd(6)-O(39)	81.1(4)
N(1)-Cd(5)-O(30)	109.7(5)	O(35)-Cd(6)-O(39)	86.9(5)
O(2)-Cd(5)-O(6)	148.2(5)	O(40)-Cd(6)-O(39)	55.0(5)
O(27)-Cd(5)-O(6)	97.8(4)	O(13)-Cd(6)-O(39)	129.9(4)
N(1)-Cd(5)-O(6)	98.5(5)	O(36)-Cd(6)-O(39)	127.1(5)
O(30)-Cd(5)-O(6)	70.8(5)	O(44)-Cd(8)-O(22)	109.4(4)
O(2)-Cd(5)-O(5)	83.8(5)	O(44)-Cd(8)-O(46)	90.8(4)
O(27)-Cd(5)-O(5)	91.7(5)	O(22)-Cd(8)-O(46)	94.5(4)
N(1)-Cd(5)-O(5)	83.1(6)	O(44)-Cd(8)-N(11)	171.3(5)
O(30)-Cd(5)-O(5)	135.6(5)	O(22)-Cd(8)-N(11)	78.4(5)
O(6)-Cd(5)-O(5)	65.2(5)	O(46)-Cd(8)-N(11)	92.5(5)
O(2)-Cd(5)-O(29)	87.9(4)	O(44)-Cd(8)-O(29)#1	88.6(4)
O(27)-Cd(5)-O(29)	82.1(4)	O(22)-Cd(8)-O(29)#1	159.4(4)
N(1)-Cd(5)-O(29)	100.1(5)	O(46)-Cd(8)-O(29)#1	95.3(4)
O(30)-Cd(5)-O(29)	51.6(4)	N(11)-Cd(8)-O(29)#1	83.1(5)
O(6)-Cd(5)-O(29)	122.5(4)	O(44)-Cd(8)-O(47)	84.2(4)
O(5)-Cd(5)-O(29)	170.7(5)	O(22)-Cd(8)-O(47)	84.8(4)
O(14)-Cd(6)-O(41)	74.2(4)	O(46)-Cd(8)-O(47)	174.3(4)
O(14)-Cd(6)-O(35)	140.7(4)	N(11)-Cd(8)-O(47)	92.8(5)
O(41)-Cd(6)-O(35)	118.6(4)	O(29)#1-Cd(8)-O(47)	87.2(4)

Table S4. Selected Bond Lengths (Å) and Angles (°) for **4**.

Tb(1)-O(2)	2.312(11)	O(2)-Tb(1)-O(60)	157.5(4)
Tb(1)-O(28)	2.319(12)	O(28)-Tb(1)-O(60)	77.3(4)
Tb(1)-O(6)	2.344(12)	O(6)-Tb(1)-O(60)	70.7(4)
Tb(1)-O(30)	2.346(13)	O(30)-Tb(1)-O(60)	87.9(4)
Tb(1)-O(61)	2.356(12)	O(61)-Tb(1)-O(60)	78.0(4)
Tb(1)-O(60)	2.384(12)	O(2)-Tb(1)-O(1)	62.9(4)
Tb(1)-O(1)	2.576(12)	O(28)-Tb(1)-O(1)	142.0(4)
Tb(1)-N(3)	2.592(16)	O(6)-Tb(1)-O(1)	77.6(4)
Tb(2)-O(68)	2.292(11)	O(30)-Tb(1)-O(1)	108.9(4)
Tb(2)-O(39)	2.312(14)	O(61)-Tb(1)-O(1)	72.0(4)
Tb(2)-O(10)	2.327(11)	O(60)-Tb(1)-O(1)	139.0(4)
Tb(2)-O(7)	2.346(11)	O(2)-Tb(1)-N(3)	73.2(4)
Tb(2)-O(38)	2.407(12)	O(28)-Tb(1)-N(3)	77.3(4)
Tb(2)-O(34)	2.412(11)	O(6)-Tb(1)-N(3)	67.4(4)
Tb(2)-N(4)	2.542(14)	O(30)-Tb(1)-N(3)	142.3(5)
Tb(2)-O(9)	2.588(12)	O(61)-Tb(1)-N(3)	140.0(5)
Cd(1)-O(2)	2.251(12)	O(60)-Tb(1)-N(3)	112.3(4)
Cd(1)-O(29)	2.252(13)	O(1)-Tb(1)-N(3)	77.1(4)
Cd(1)-N(1)	2.310(14)	O(68)-Tb(2)-O(39)	76.5(5)
Cd(1)-O(27)	2.338(11)	O(68)-Tb(2)-O(10)	82.8(4)
Cd(1)-O(31)	2.360(11)	O(39)-Tb(2)-O(10)	78.8(4)
Cd(1)-O(33)	2.371(12)	O(68)-Tb(2)-O(7)	118.4(4)
Cd(2)-O(35)	2.218(15)	O(39)-Tb(2)-O(7)	147.3(5)
Cd(2)-O(3)	2.232(12)	O(10)-Tb(2)-O(7)	129.4(4)
Cd(2)-O(37)	2.259(13)	O(68)-Tb(2)-O(38)	142.4(4)
Cd(2)-O(32)	2.271(15)	O(39)-Tb(2)-O(38)	73.1(5)
Cd(2)-O(31)	2.361(13)	O(10)-Tb(2)-O(38)	111.8(4)
Cd(2)-O(4)	2.384(16)	O(7)-Tb(2)-O(38)	79.8(4)
Cd(2)-O(36)	2.63(2)	O(68)-Tb(2)-O(34)	78.1(4)
Cd(3)-O(3)	2.185(13)	O(39)-Tb(2)-O(34)	86.1(4)
Cd(3)-N(2)	2.213(16)	O(10)-Tb(2)-O(34)	158.0(4)
Cd(3)-O(37)	2.275(13)	O(7)-Tb(2)-O(34)	70.5(4)
Cd(3)-O(7)	2.280(11)	O(38)-Tb(2)-O(34)	78.2(4)
Cd(3)-O(34)	2.303(11)	O(68)-Tb(2)-N(4)	77.9(4)
Cd(3)-O(8)	2.533(11)	O(39)-Tb(2)-N(4)	143.5(5)
Cd(3)-O(33)	2.631(11)	O(10)-Tb(2)-N(4)	72.7(4)
Cd(4)-O(40)	2.240(13)	O(7)-Tb(2)-N(4)	68.7(4)
Cd(4)-O(10)	2.260(11)	O(38)-Tb(2)-N(4)	138.8(4)
Cd(4)-O(67)	2.269(11)	O(34)-Tb(2)-N(4)	113.4(4)
Cd(4)-O(43)	2.316(12)	O(68)-Tb(2)-O(9)	141.4(4)
Cd(4)-N(5)	2.346(15)	O(39)-Tb(2)-O(9)	109.8(4)
Cd(4)-O(41)	2.353(11)	O(10)-Tb(2)-O(9)	62.3(4)
O(2)-Tb(1)-O(28)	83.0(4)	O(7)-Tb(2)-O(9)	77.5(4)
O(2)-Tb(1)-O(6)	129.1(4)	O(38)-Tb(2)-O(9)	71.3(4)
O(28)-Tb(1)-O(6)	116.8(4)	O(34)-Tb(2)-O(9)	138.9(4)
O(2)-Tb(1)-O(30)	76.9(4)	N(4)-Tb(2)-O(9)	76.3(4)
O(28)-Tb(1)-O(30)	76.7(4)	O(2)-Cd(1)-O(29)	107.6(4)
O(6)-Tb(1)-O(30)	149.9(4)	O(2)-Cd(1)-N(1)	77.5(5)
O(2)-Tb(1)-O(61)	112.4(4)	O(29)-Cd(1)-N(1)	174.1(5)
O(28)-Tb(1)-O(61)	141.5(4)	O(2)-Cd(1)-O(27)	96.4(4)
O(6)-Tb(1)-O(61)	81.5(4)	O(29)-Cd(1)-O(27)	93.4(4)
O(30)-Tb(1)-O(61)	73.3(4)	N(1)-Cd(1)-O(27)	88.9(4)

O(2)-Cd(1)-O(31)	84.8(4)	O(3)-Cd(3)-O(7)	148.5(5)
O(29)-Cd(1)-O(31)	84.3(4)	N(2)-Cd(3)-O(7)	97.3(5)
N(1)-Cd(1)-O(31)	93.4(5)	O(37)-Cd(3)-O(7)	98.5(4)
O(27)-Cd(1)-O(31)	177.6(4)	O(3)-Cd(3)-O(34)	136.0(4)
O(2)-Cd(1)-O(33)	161.1(4)	N(2)-Cd(3)-O(34)	107.1(5)
O(29)-Cd(1)-O(33)	88.2(4)	O(37)-Cd(3)-O(34)	89.1(4)
N(1)-Cd(1)-O(33)	86.2(4)	O(7)-Cd(3)-O(34)	73.7(4)
O(27)-Cd(1)-O(33)	92.8(4)	O(3)-Cd(3)-O(8)	82.7(4)
O(31)-Cd(1)-O(33)	86.6(4)	N(2)-Cd(3)-O(8)	89.9(5)
O(35)-Cd(2)-O(3)	104.3(6)	O(37)-Cd(3)-O(8)	85.4(4)
O(35)-Cd(2)-O(37)	113.3(5)	O(7)-Cd(3)-O(8)	65.8(4)
O(3)-Cd(2)-O(37)	75.2(5)	O(34)-Cd(3)-O(8)	137.7(4)
O(35)-Cd(2)-O(32)	97.5(5)	O(3)-Cd(3)-O(33)	85.8(4)
O(3)-Cd(2)-O(32)	146.2(5)	N(2)-Cd(3)-O(33)	102.5(5)
O(37)-Cd(2)-O(32)	119.2(5)	O(37)-Cd(3)-O(33)	78.5(4)
O(35)-Cd(2)-O(31)	151.8(5)	O(7)-Cd(3)-O(33)	124.0(4)
O(3)-Cd(2)-O(31)	95.5(4)	O(34)-Cd(3)-O(33)	50.6(4)
O(37)-Cd(2)-O(31)	90.9(4)	O(8)-Cd(3)-O(33)	162.1(4)
O(32)-Cd(2)-O(31)	56.4(4)	O(40)-Cd(4)-O(10)	108.6(4)
O(35)-Cd(2)-O(4)	80.5(6)	O(40)-Cd(4)-O(67)	94.2(4)
O(3)-Cd(2)-O(4)	68.0(5)	O(10)-Cd(4)-O(67)	95.1(4)
O(37)-Cd(2)-O(4)	142.9(5)	O(40)-Cd(4)-O(43)	88.9(5)
O(32)-Cd(2)-O(4)	90.9(5)	O(10)-Cd(4)-O(43)	160.3(4)
O(31)-Cd(2)-O(4)	88.7(5)	O(67)-Cd(4)-O(43)	92.3(4)
O(35)-Cd(2)-O(36)	51.9(6)	O(40)-Cd(4)-N(5)	172.4(5)
O(3)-Cd(2)-O(36)	131.2(6)	O(10)-Cd(4)-N(5)	78.1(5)
O(37)-Cd(2)-O(36)	78.3(5)	O(67)-Cd(4)-N(5)	88.6(4)
O(32)-Cd(2)-O(36)	82.6(6)	O(43)-Cd(4)-N(5)	83.9(5)
O(31)-Cd(2)-O(36)	125.3(5)	O(40)-Cd(4)-O(41)	84.6(5)
O(4)-Cd(2)-O(36)	130.2(6)	O(10)-Cd(4)-O(41)	83.6(4)
O(3)-Cd(3)-N(2)	84.4(5)	O(67)-Cd(4)-O(41)	177.9(4)
O(3)-Cd(3)-O(37)	75.8(5)	O(43)-Cd(4)-O(41)	89.5(4)
N(2)-Cd(3)-O(37)	160.0(5)	N(5)-Cd(4)-O(41)	92.8(5)
